# Sleep parameters and problems in adolescents with and without ADHD: A systematic review and meta‐analysis

**DOI:** 10.1002/jcv2.12151

**Published:** 2023-03-11

**Authors:** Finja Marten, Lena Keuppens, Dieter Baeyens, Bianca E. Boyer, Marina Danckaerts, Samuele Cortese, Saskia Van der Oord

**Affiliations:** ^1^ KU Leuven Leuven Belgium; ^2^ Leiden University Leiden The Netherlands; ^3^ Center for Innovation in Mental Health School of Psychology Faculty of Environmental and Life Sciences University of Southampton Southampton UK; ^4^ Clinical and Experimental Sciences (CNS and Psychiatry) Faculty of Medicine University of Southampton Southampton UK; ^5^ Solent NHS Trust Southampton UK; ^6^ Hassenfeld Children's Hospital at NYU Langone New York University Child Study Center New York City New York USA; ^7^ Division of Psychiatry and Applied Psychology School of Medicine University of Nottingham Nottingham UK

**Keywords:** ADHD, adolescence, sleep

## Abstract

**Background:**

Adolescence is characterized by an increase in the rate of sleep problems, which might be even more pronounced in adolescents with ADHD. This systematic review with meta‐analysis aimed to compare sleep in adolescents with and without ADHD, including sleep parameters, both subjectively and objectively measured, sleep problems and sleep hygiene.

**Methods:**

Medline, CINAHL, PsycINFO, EMBASE, ERIC, Web of Science, and PubMed databases were searched for studies with case‐control designs (published between 1980 and 2022) directly comparing sleep in adolescents (12–25 years) with ADHD to typically developing controls. Standardized mean differences were calculated and a random‐effects model was implemented using RevMan.

**Results:**

Overall, 6974 titles/abstracts and 205 full texts were screened, resulting in 13 eligible studies. The sample sizes range from 35 to 9846 with in total 2465 adolescents with ADHD and 18,417 controls. The data suggests that adolescents with ADHD report significantly more disturbed subjective sleep parameters (e.g., total sleep time; *n* = 7, SMD = 0.47, *p* < .001) and experience more sleep problems compared to typically developing peers (e.g., daytime sleepiness; *n* = 5, SMD = 0.54, *p* = .01). Only few studies objectively measured sleep and no significant differences were found between both groups (*n* = 3) in any parameter. Differences in sleep hygiene could not be examined due to a limited number of studies.

**Conclusions:**

Adolescents with ADHD report significantly worsened subjectively sleep parameters and more sleep problems compared to controls. These findings are still preliminary as a limited number of studies was identified. Nevertheless, it is advised to routinely include sleep assessment in the ADHD diagnostic process. More research is needed with a focus on objective measurement and sleep hygiene in ADHD.

## Graphical abstract

The present meta‐analytic review compares sleep parameters and problems in adolescents with ADHD to their typically developing peers. It suggests that subjective sleep parameters are more disturbed in adolescents with ADHD and that they experience more sleep problems.

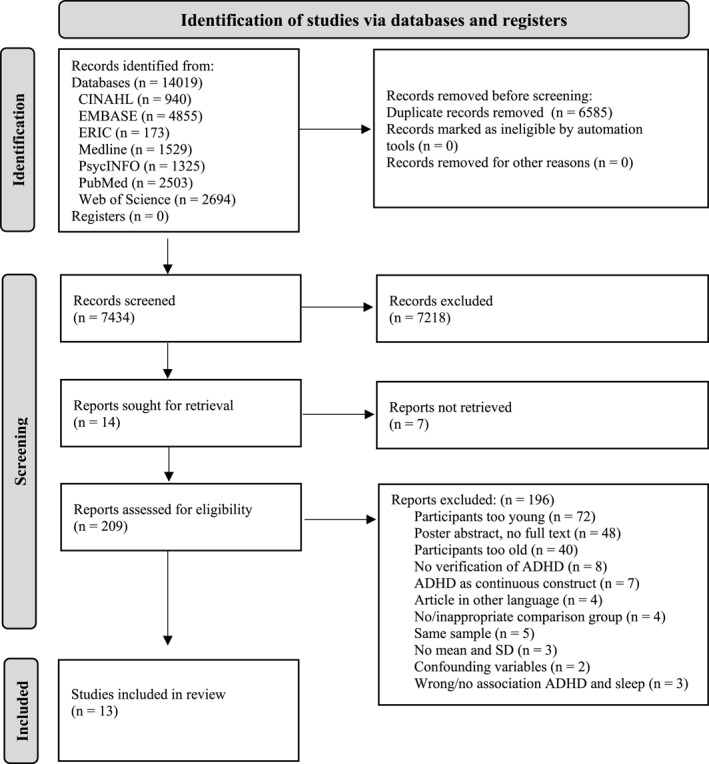

**Key points**

- This meta‐analytic review is the first to compare the sleep of adolescents with and without ADHD, thereby distinguishing between sleep parameters, measured both subjectively and objectively, sleep problems, and sleep hygiene.
- Results suggest that adolescents with ADHD report significantly worsened subjective sleep parameters and significantly more sleep problems compared to their typically developing peers.
- These findings highlight the importance of routinely assessing sleep in the ADHD diagnostic process.
- More research in adolescents with ADHD is needed with a focus on objective measurement to get the full picture and on sleep hygiene as this might be one of the main modifiable variables to improve their sleep.

## INTRODUCTION

In the last centuries, the definition of adolescence has changed and adolescence is now considered to last until mid 20s (Sawyer et al., 2018), as the brain continues to develop higher‐level capacities up to mid/late 20s (Wood et al., 2018). This developmental phase is characterized by major changes in sleep, including a slower buildup of sleep pressure and a delayed sleep‐wake timing (Carskadon & Tarokh, 2013). Furthermore, various behaviors that are more common in adolescence significantly affect sleep (alcohol, caffeine, social media use). These biological and environmental changes in adolescence result in a wide array of sleep problems, which in turn negatively influence daily life.

The study of sleep encompasses a diversity of factors including sleep parameters, sleep problems, and sleep hygiene. Sleep parameters are the basic structure of sleeping patterns, such as total sleep time (TST). Sleep problems are “nonspecific sleep‐related complaints” (Cortese et al., 2013), often associated with mutually exacerbating conditions, such as psychiatric comorbidities. Sleep hygiene refers to the sleep environment, sleep practices, and physiological factors such as caffeine (Martin et al., 2020). Research suggests that insufficient sleep leads to a range of negative outcomes, including behavioral problems (Meijer et al., 2010) and lower grades in school (Curcio et al., 2006). These sleep problems and related behaviors might be even more pronounced in adolescents with Attention‐Deficit/Hyperactivity Disorder (ADHD; Becker, Langberg et al., 2019; Lunsford‐Avery et al., 2016). ADHD, characterized by inattention, hyperactivity and/or impulsivity, causes significant impairments throughout the lifespan (American Psychiatric Association, 2013), and it has been estimated that up to 72% of adolescents with ADHD present with sleep problems (Langberg et al., 2017). Factors underlying these sleep problems in adolescents with ADHD are still not fully understood. However, there is some research suggesting factors such as inappropriate sleep hygiene, academic pressure, comorbid mental health problems, and medication use (Becker, 2019). Based on two sleep restriction studies, shortened sleep has in turn been found to be causally related to increased attention problems, increased oppositional symptomatology (Becker, Epstein et al., 2019) and more depressive symptoms (Becker et al., 2020). Thus, sleep problems and ADHD symptomatology seem to be bi‐directionally related, resulting in a vicious circle of sleep problems and impairment.

So far, one systematic review has examined sleep problems in adolescents with ADHD. They concluded that sleep problems are prevalent in adolescents with ADHD and are related to worsened clinical, neurocognitive and functional outcomes (Lunsford‐Avery et al., 2016). However, as they included studies solely including adolescents with ADHD, comparison of sleep with typically developing (TD) controls is difficult. Additionally, a number of studies were published since then, comparing sleep in adolescents with and without ADHD (e.g., Becker et al., 2021; Becker et al., 2019; Cadman et al., 2016; Frick et al., 2022; Gregory et al., 2017; Liu et al., 2020; Ng et al., 2017; Takahashi et al., 2016; Zerón‐Rugerio et al., 2021). To provide a detailed assessment of sleep in adolescents with and without ADHD, sleep should be measured both subjectively (sleep diaries, questionnaires) and objectively (polysomnography (PSG), actigraphy), as findings have been non‐overlapping (Becker, Langberg et al., 2019). Therefore, there is a clear need for a recent systematic meta‐analysis comparing multiple aspects of sleep in adolescents with and without ADHD.

Therefore, the current meta‐analytic review aimed at examining the sleep of adolescents with and without ADHD. A comparison was made in relation to (i) sleep parameters, thereby making a distinction between subjective and objective measurements, (ii) sleep problems, and (iii) sleep hygiene.

## METHODS

### Search strategy

The protocol of this meta‐analytic review was registered at PROSPERO (registration number CDR42020181689, available from: https://www.crd.york.ac.uk/PROSPERO/display_record.php?RecordID=181689) and PRISMA guidelines (Page et al., 2020) were followed. The search was conducted using Medline, CINAHL, PsycINFO, EMBASE, ERIC, Web of Science, and PubMed databases for articles added between first of January 1980 and 21st of March 2022. The search term was developed using Medical Subject Headings (MeSH) and Boolean operators: ADHD OR attention deficit hyperactivity disorder AND adolescence OR teen OR youth OR young adult OR child OR adult OR student OR high school AND sleep OR insomnia OR circadian rhythm. Additionally, references of included studies and the one previous review (Lunsford‐Avery et al., 2016) were screened for other eligible studies. Peer‐reviewed publications in English, Dutch, and German were included.

### Inclusion and exclusion criteria

Inclusion criteria were (a) case‐control designs, (b) fulfilled ADHD criteria in the ADHD group, confirmed by the researchers based on DSM criteria (from DSM III onwards) or ICD criteria (from ICD‐10 onwards), and (c) one of two age criteria had to be met. Either, (i) all participants of the study had to be in the age range of 12–25 years, or (ii) the standard deviation both added to and subtracted from the study's mean age, had to be in the range between 12 and 25 years. Exclusion criteria were (a) studies aimed at specific samples with comorbid ADHD (e.g., ADHD and comorbid depression) and (b) single case designs.

### Study selection and data extraction

Titles and abstracts, followed by full papers, were screened independently by the first two authors (FM/LK). In case of disagreement, research group members were consulted (DB/BB/MD/SVDO). Data was independently extracted from the papers (FM/LK), and if necessary requested from the corresponding authors. Retrieved data included study information (authors, publication year, location, country), participant details (study size, age, gender, subtype/presentation of ADHD, medication), and sleep measures (mean and SD).

### Outcomes

The following sleep domains were assessed; (a) subjective and objective sleep parameters, (b) sleep problems and (c) sleep hygiene. Sleep parameters were defined as any subjective sleep parameter from sleep diaries or questionnaires and any objective sleep parameter as assessed by actigraphy or PSG. Sleep problems included any subjective problem with timing, amount, and quality of sleep, based on questionnaires. For sleep hygiene, the current meta‐analytic review required that the effect of the sleep hygiene variable on sleep was described. As suggested by Valentine et al. (2010), outcome domains being reported by at least two studies were considered in the meta‐analytic review (Table 1).

**TABLE 1:** Sleep outcomes included in the meta‐analysis.

Outcomes	Description
Subjective sleep parameters	
Total sleep time	Duration of total sleep at night.
Sleep efficiency	Ratio of total sleep time to nocturnal time in bed.
Sleep onset latency	The time from lights off to sleep onset.
Wake after sleep onset	The time of being awake during the night.
Bedtime	The time of going to bed or turning off the lights.
Wake time	The time of waking up in the morning.
Number of awakenings	The number of awakenings during the night.
Objective sleep parameters	
Total sleep time	Duration of total sleep at night.
Sleep efficiency	Ratio of total sleep time to nocturnal time in bed.
Wake after sleep onset	The time of being awake during the night.
Sleep onset time	Point at which adolescent falls asleep.
Sleep offset time	Point at which adolescent wakes up.
Sleep problems	
Insomnia	Difficulties falling asleep, staying asleep and waking up too early in the morning without being able to fall asleep again.
Daytime sleepiness	A persistent tiredness and lack of energy during the day.
Sleep disturbances	All sleep disorders/problems, without distinction of type.
Sleep satisfaction	Satisfaction with daily sleep.

### Statistical analyses

Analyses were performed using Review Manager 5.4 (The Cochrane Collaboration, 2019). First, study quality was independently evaluated (FM/LK), using the Newcastle‐Ottawa Scale for assessing the quality of nonrandomized studies (NOS). A total score of less than five was considered poor, of at least five fair, and of seven or higher good (Wells et al., n.d.). Disagreements were resolved through discussion and if necessary, by consultation of the research group. Sensitivity analyses examined the impact of poor studies. Second, outcomes were categorized into one of the outcome domains, based on descriptions of (sub)scales measuring the specific concept or if not available on a comparison of the individual items of questionnaires. We calculated the weighted average of mean and standard deviation for the ADHD group when separate ADHD subtype/presentation scores were reported (Bauermann, 2010). Additionally, when outcomes were reported separately for week and weekend (Becker et al., 2021; Becker, Langberg et al., 2019; Hysing et al., 2016; Voinecscu et al., 2012), each outcome was examined separately and weighted averages of mean and standard deviation were calculated combining week and weekend. When multiple informant ratings of one concept were available, the informant of which most ratings were already reported was chosen to reduce variability within outcomes (Becker, Langberg et al., 2019).

Standardized mean differences (SMD) were calculated for sleep parameters and sleep problems. For sleep hygiene, correlation coefficients of the sleep hygiene variable and sleep outcome from both groups were compared. Based on the expectation that effects vary across studies and that there is sampling variability (Riley et al., 2011), a random‐effects model was chosen. Effect sizes up to 0.5 were considered to be small, up to 0.8 were medium, and above 0.8 large. The heterogeneity of effect sizes was calculated a posteriori by the I^2^ index and a 95% confidence interval, providing a more reliable estimation of heterogeneity in case of limited studies (von Hippel, 2015). Funnel plots and Egger tests for funnel plot asymmetry, evaluating publication bias, were assessed when there were at least 10 studies per outcome (Sterne et al., 2011). Meta‐regression, testing the effect of other moderators including ADHD subtype/presentation, medication use, age, and gender, was executed in case of more than 10 studies (Higgins et al., 2019). In case of less than 10 studies sensitivity analyses were done for medication use, as ADHD medication may impact sleep (Becker, 2019), and age thereby solely including studies of late adolescence (minimum inclusion age of 18 years; de Wit & van Aken, 2019).

## RESULTS

### Study selection and characteristics

**FIGURE 1:**
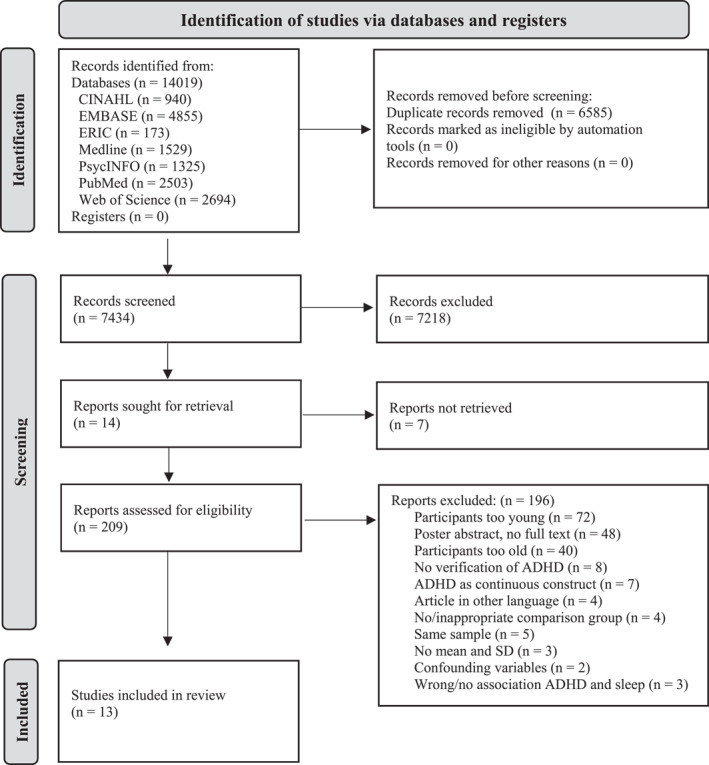
Flowchart of selection process. Flowchart of the search results (performed July 2020, updated March 2022). See Table S2 for specific reasons for exclusion.

Overall, 7974 titles and abstracts and 209 full texts were screened, resulting in 15 eligible studies of which three studies reported on the same sample (Figure 1). Data was extracted from the most relevant report (Becker, Langberg et al., 2019). A sensitivity analysis was conducted without the study by Ng et al. (2017) as they had a sample of 50 participants with a previous ADHD diagnosis in the ADHD group, but only 45 scoring high enough on the questionnaire measuring current ADHD symptoms. Thirteen reports were included in the meta‐analysis, with data being extracted from the papers (*n* = 6) or by contacting authors (*n* = 7).

A minority of studies looked at daily fluctuations of sleep; two combined actigraphy and sleep diaries, and one only used actigraphy. Despite one study, all studies used subjective measurements. The study designs included case‐control studies, cohort studies and cross‐sectional studies. The sample sizes of the studies included ranged from 35 to 9846 with a total of 2465 adolescents with ADHD and 18,417 controls (Table 2). Approximately half of the participants was male (49.63%) and the average age was 17.30 years (SD = 1.45 years). Subjects were recruited through multiple resources.

**TABLE 2:** Characteristics of included studies.

Study	Location	Study design	ADHD subtype	NOS	ADHD	TD	Age	Male%	Medication ADHD	Subjective parameter	Objective parameter	Sleep problem
					*N*	*N*						
Bauermann (2010)	CA	Case‐control	All	Fair	147	222	20.80 (4.82)	34	No info			SPI
Becker et al. (2021)	US	Case‐control	All	Fair	58	64	15–17	61	83% ADHD medication	Self‐reported		
Becker, Langberg et al. (2019)	US	Case‐control	All	Good	162	140	12–14	55	All ADHD medication	Sleep diary	Actigraphy	SSHS
Cadman et al. (2016)	GB	Case‐control cohort	All	Fair	85	221	14–23	84	No info			CIS‐R
Frick et al. (2022)	SE	Case‐control	All	Poor	180	103	13–19	33.2	68.9% ADHD medication			KSQ
Gregory et al. (2017)	GB	Cohort	All	Good	54	1683	18	49	24% ADHD medication			PSQI
Hysing et al. (2016)	NO	Case‐control	All	Good	1023	8823	17	47	No info	Self‐reported		Self‐reported
Liu et al. (2020)	CN	Cohort	All	Good	541	6531	14.59 (1.45)	50	No info	AHQ		AHQ
Mullin et al. (2011)	US	Case‐control	Combined	Fair	14	21	11–17	62	1 day wash‐out for ADHD medication	Sleep diary	Actigraphy	
Ng et al. (2017)	CN	Case‐control	All	Good	52	50	21.20 (1.28)	100	13% ADHD medication	Self‐reported		
Takahashi et al. (2016)	JP	Cross‐sectional	All	Poor	101	270	18–22	0	No info			Self‐reported
Voinescu et al. (2012)	RO	Case‐control	All	Fair	30	271	21.82 (2.52)	15	No info	CSM, STQ		SDQ, SCI
Zerón‐Rugerio et al. (2021)	ES	Case‐control	All	Good	18	18	12–16	55	No ADHD medication		Actigraphy	

Abbreviations: AHQ, Adolescent Health Questionnaire; CA, Canada; CIS‐R, Clinical Interview Schedule‐Revised; CN, China; CSM, Composite Scale of Morningness; ES, Spain; GB, Great Britain; JP, Japan; KSQ, Karolinska Sleep Questionnaire; NO, Norway; NOS, Newcastle Ottawa Scale; PSQI, Pittsburgh Sleep Quality Index; RO, Romania; SCI, Sleep Condition Indicator; SDQ, Sleep Disorders Questionnaire; SE, Sweden; SPI, Sleep Problems Inventory; SSHS, School Sleep Habits Survey; STQ, Sleep Timing Questionnaire; US, United States of America.

### Risk of bias and publication bias

Regarding study quality, six studies had a good qualification, five a fair score and two were poor (Table S1; interrater reliability; *κ* = 0.85). The most common subdomain on which study quality was low, was the exposure (i.e., assessment of outcomes). Sensitivity analyses without the two studies with poor quality showed that excluding the study of Frick et al. (2022) resulted in similar effects. However, when excluding the study of Takahashi et al. (2016), adolescents with ADHD experienced significantly more insomnia than TD adolescents. Publication bias was not assessed as a limited number of studies were included.

### Subjective sleep parameters

**FIGURE 2:**
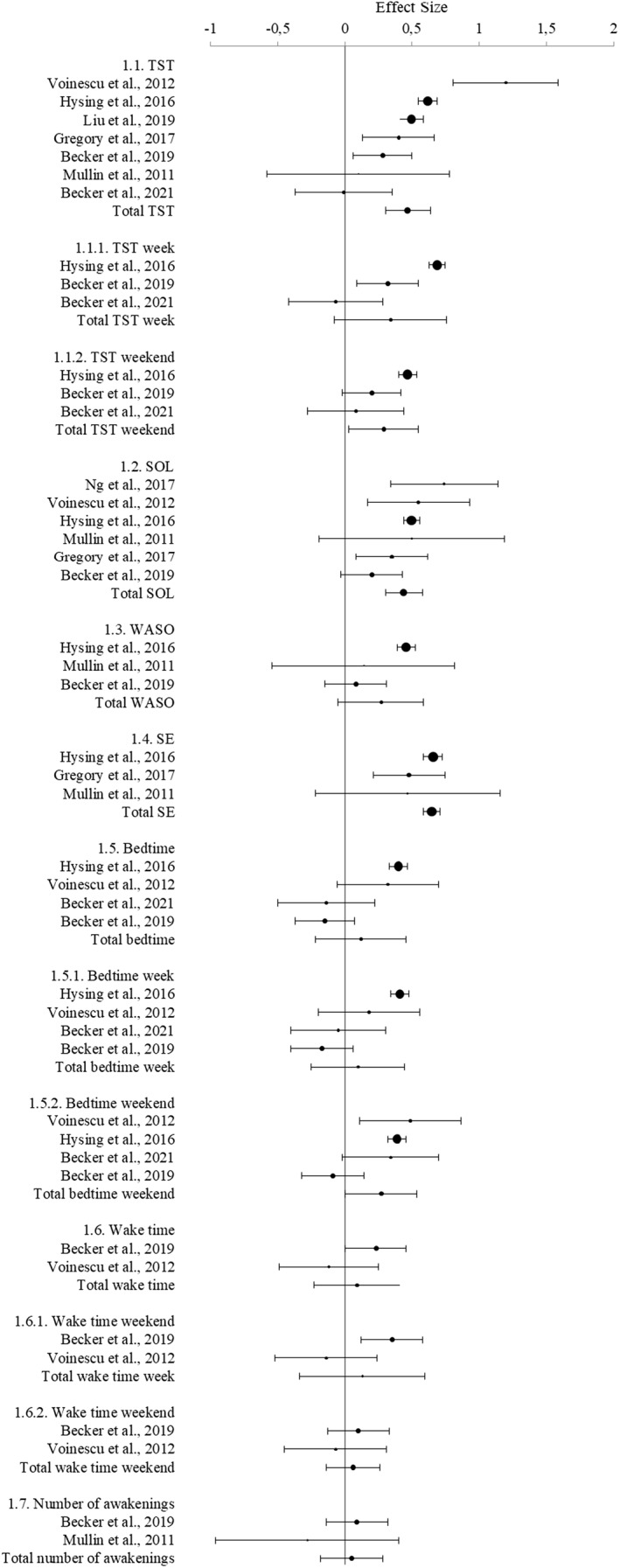
Forest plot showing the meta‐analysis of subjective sleep parameters.

Nine studies examined subjective sleep variables, some reported multiple variables (Table 3, Figure 2). TST was significantly shorter in the ADHD group, but when distinguishing between week and weekend nights, only weekend nights were significant, and all had significant heterogeneity. SOL was significantly longer in adolescents with ADHD, without heterogeneity impacting the results. Moreover, SE ratings in the ADHD group were significantly lower, without a significant impact of heterogeneity. Bedtime showed a trend towards significance with being later in the ADHD group, during the weekend, and this was significantly impacted by heterogeneity. TST during the week, wake after sleep onset, bedtime and bedtime during the week, wake time (week and weekend), and number of awakenings did not significantly differ between the two groups. Sensitivity analyses suggested that both age and medication might impact the results (Table S3).

**TABLE 3:** Results of the meta‐analysis related to subjective sleep parameters.

Subjective sleep parameters (*k*)	ADHD (*n*)	Control (*n*)	SMD (95% CI)	*z*	*p*	Effect size	Heterogeneity	
							*I* ^2^ (%)	*p*
TST (7)	1882	1753	0.47 (0.31 to 0.64)	5.59	<.001	Small	83	<.001
TST week (3)	1243	9027	0.34 (−0.07 to 0.76)	1.62	.1		92	<.001
TST weekend (3)	1243	9027	0.29 (0.3 to 0.55)	2.2	<.001	Small	79	<.01
SOL (6)	1335	10,988	0.44 (0.3 to 0.58)	6.22	<.001	Small	43	.12
WASO (3)	1199	8984	0.29 (−0.5 to 0.59)	1.64	.1		81	<.01
SE (3)	1091	10,527	0.65 (0.59 to 0.71)	20.15	<.001	Medium	0	.4
Bedtime (4)	1273	9298	0.12 (−0.23 to 0.46)	0.66	.51		89	<.001
Bedtime week (4)	1273	9298	0.1 (−0.25 to 0.46)	0.57	.57		90	<.001
Bedtime weekend (4)	1273	9298	0.27 (0.00 to 0.54)	1.99	.05	Small	82	.001
Waketime (2)	192	411	0.09 (−0.24 to 0.43)	0.54	.59		59	.12
Waketime week (2)	192	411	0.13 (−0.35 to 0.6)	0.53	.6		79	.03
Waketime weekend (2)	192	411	0.06 (−0.14 to 0.25)	0.59	.56		0	.44
Number of awakenings (2)	176	161	0.05 (−0.18 to 0.28)	0.49	.67		3	.31

### Objective sleep parameters

**FIGURE 3:**
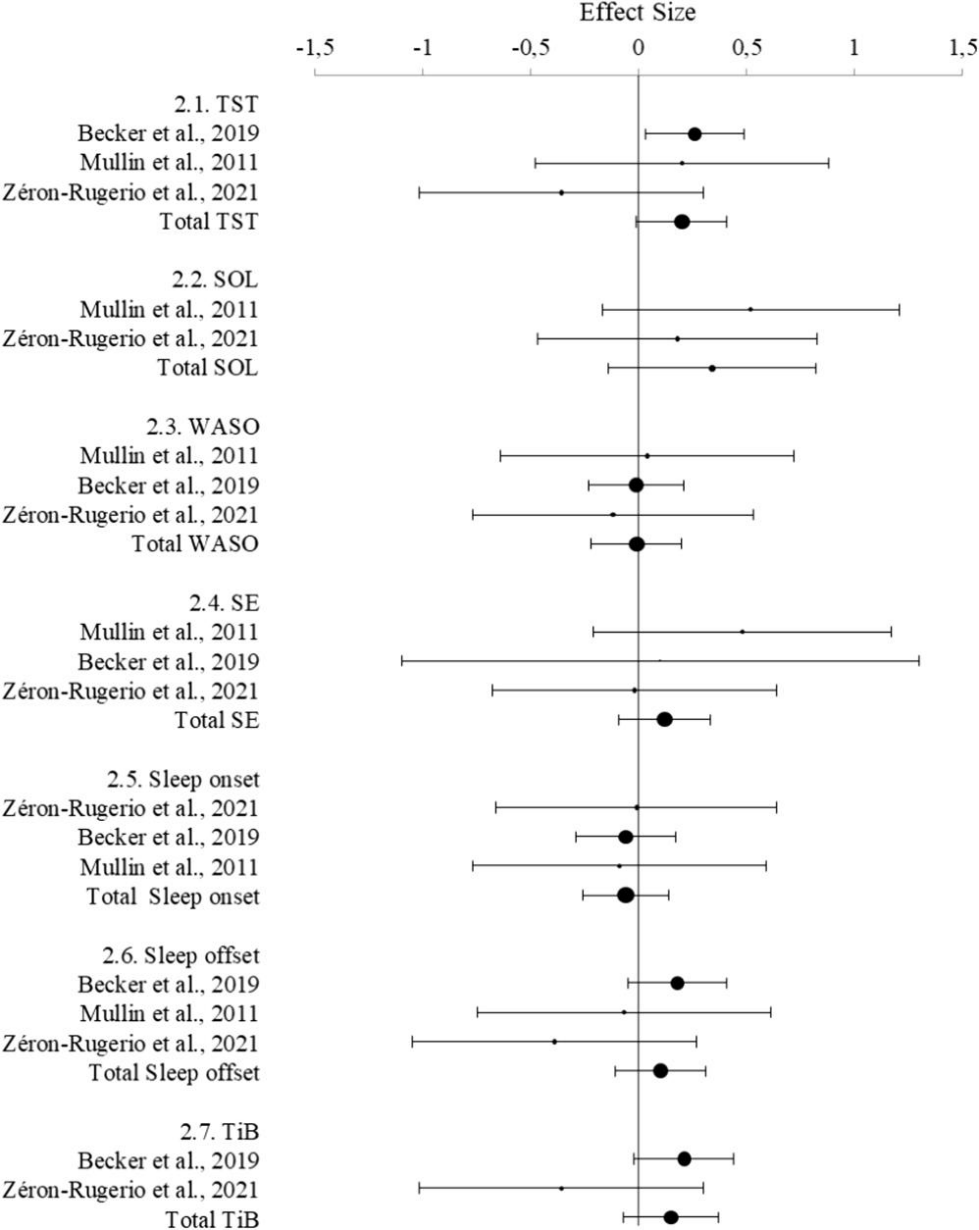
Forest plot showing the meta‐analysis of objective sleep parameters.

Only three studies examined objective sleep parameters (Table 4, Figure 3). No differences between groups on objectively measured sleep parameters were significant. Due to a limited number of studies sensitivity analyses were not conducted.

**TABLE 4:** Results of the meta‐analysis related to objective sleep parameters.

Objective sleep parameters (*k*)	ADHD (*n*)	Control (*n*)	SMD (95% CI)	*z*	*p*	Heterogeneity	
						*I* ^2^ (%)	*p*
TST (3)	194	179	0.13 (−0.21 to 0.47)	0.74	.46	34	.22
SOL (2)	32	39	0.34 (−0.13 to 0.82)	1.42	.16	0	.48
WASO (3)	194	179	0.01 (−0.19 to 0.22)	0.13	.9	0	.94
SE (3)	194	179	0.12 (−0.08 to 0.33)	1.20	.23	0	.53
Sleep onset (3)	194	179	0.06 (−0.14 to 0.26)	0.59	.56	0	.99
Sleep offset (3)	194	179	0.3 (−0.29 to 0.35)	0.18	.86	30	.24
TiB (2)	180	158	0.1 (−0.53 to 0.55)	0.04	.97	62	.11

### Sleep problems

**FIGURE 4:**
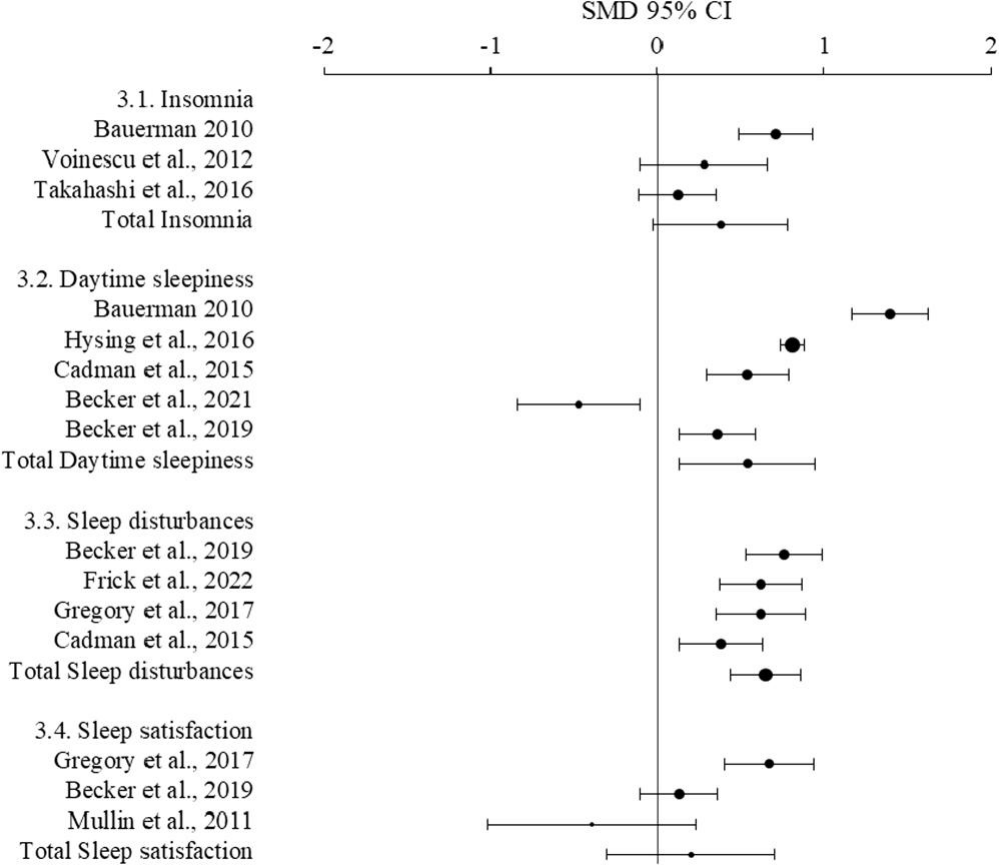
Forest plot showing the meta‐analysis of sleep problems.

Four studies reported sleep problems, all based on adolescent report (Table 5, Figure 4). Adolescents with ADHD reported significantly more daytime sleepiness and more sleep disturbances. Insomnia was not significantly different between the groups, however, when excluding the study with poor quality, it was. Subjective satisfaction was not significant. Sensitivity analyses suggested that age does not impact the results, but medication might (Table S3).

**TABLE 5:** Results of the meta‐analysis related to sleep problems.

Sleep problems (*k*)	ADHD (*n*)	Control (*n*)	SMD (95% CI)	*z*	*p*	Effect size	Heterogeneity	
							*I* ^2^ (%)	*p*
Insomnia (3)	278	763	0.38 (−0.03–0.78)	1.82	.07		86	<.001
Daytime sleepiness (4)	1417	9406	0.54 (0.13–0.96)	2.56	.01	Medium	96	<.001
Sleep disturbances (5)	499	2165	0.65 (0.44–0.86)	6.12	<.001	Medium	61	.04
Subjective satisfaction (3)	230	1864	0.2 (−0.31–0.7)	0.76	.45		86	<.001
Sensitivity analysis without Takahashi								
Insomnia (2)^a^	177	493	0.53 (0.11–0.94)	2.49	.01	Medium	73	.05

^a^ Sensitivity analysis without Takahashi et al. (2016)

### Sleep hygiene

Only two studies reported a link between the sleep hygiene behavior and sleep, assessing different sleep hygiene related behaviors (i.e. caffeine (Cusick et al., 2020)/technology use (Bourchtein et al., 2019)) making a meta‐analysis impossible, therefore this outcome is not further included in this study.

## DISCUSSION

The present study is the first meta‐analytic review comparing sleep parameters and problems in adolescents with ADHD to their TD peers. It suggests that some subjective sleep parameters are more disturbed in adolescents with ADHD and that they seem to experience more sleep problems, but do not differ on objective sleep parameters. These findings however, are rather preliminary, as a limited number of studies could be included. No conclusions can be made regarding their sleep hygiene, due to a lack of studies.

This meta‐analytic review suggests that subjectively measured sleep might be more disturbed in adolescents with ADHD. This is in line with both a meta‐analysis about sleep in children (Cortese et al., 2009) and in adults (Díaz‐Román et al., 2018). Both found the sleep of individuals with ADHD to be subjectively more disturbed compared to TD controls, suggesting that this may be a lifelong problem. Current results suggest that this might be influenced by age, as older adolescents show more disturbed sleep. Regarding the effect of heterogeneity, most subjective results were impacted by this. Consequently, they have to be interpreted with caution. The only two variables without significant heterogeneity were SOL and SE, suggesting that these are worse in adolescents with ADHD across studies. Regarding objective measurements, we found no significant differences. This is in contrast with the subjective findings, but again partially in line with the two previous meta‐analyses examining sleep in children (Cortese et al., 2009) and adults with and without ADHD (Díaz‐Román et al., 2018).

Several factors may account for the subjectively more disturbed sleep in adolescents with ADHD. First, although there is a lack of studies the few existing studies indicate that adolescents with ADHD may portray more inappropriate sleep hygiene behaviors than TD adolescents, which has been shown to correlate with a shorter TiB (Bourchtein et al., 2019) and more sleep problems (Martin et al., 2020). Second, adolescents with ADHD may have a more variable sleep/wake pattern (Langberg et al., 2019). This could result in more ill‐timed sleep and consequently, a lack of sleep. Third, adolescents experience enhanced academic pressure, especially during the school week, delaying their sleep schedule (Carskadon, 2011). This might be even more influential in adolescents with ADHD due to their underlying difficulties with planning, organization, and time management (Boyer et al., 2018). Fourth, adolescents with ADHD often take stimulant medication. Previous studies have shown that ADHD medication impacts sleep parameters including shorter sleep duration and later sleep onset times (Santisteban et al., 2014). Most studies in this meta‐analysis did not report whether participants used medication, however, based on sensitivity analyses with three studies, this meta‐analysis suggests that medication might decrease differences in subjective sleep parameters between adolescents with and without ADHD, but does not seem to have an effect on sleep problems. These findings are rather ambiguous and based on few studies, and consequently, no conclusions can be made.

Considering sleep problems, adolescents with ADHD showed higher rates of daytime sleepiness and sleep disturbances than TD peers. Daytime sleepiness was also supported by teachers rating adolescents with ADHD to experience more sleepiness during the day than controls (Becker, Langberg et al., 2019). This is especially concerning, due to the associations of daytime sleepiness with increased ADHD symptoms (Sivertsen et al., 2015). Findings on insomnia were only significant when doing sensitivity analyses without the study with poor quality (without Takahashi et al., 2016). This raises the question, whether studies with poor quality should be excluded in meta‐analyses, as these may distort the results. However, this is not supported by the exclusion of the second study with poor quality (Frick et al., 2022), which did not affect the results. Consequently, we suggest that analyses should be conducted with and without the studies with poor quality, to examine whether these might impact the findings.

Some limitations can be mentioned. First, albeit the importance of sleep hygiene for sleep (Martin et al., 2020), this outcome could not be included in the current meta‐analytic review and no conclusions can be made regarding its role in the sleep of adolescents with ADHD. Second, only a limited number of studies were identified and included. Especially studies using objective measurements were lacking. Consequently, funnel plots and meta‐regression could not be conducted. Third, the studies included differed considerably in sample sizes and inclusion criteria. Nevertheless, we still found significant differences between the two groups, suggesting that the more disturbed sleep might also be a problem in adolescents that merely portray ADHD symptoms or are not (fully) diagnosed. Fourth, included studies used different operationalizations for most subjective sleep variables. Notwithstanding the clear distinction in our meta‐analytic review between different sleep domains, we did find significant heterogeneity for most of our outcomes, which might be partially due to the different measurements/questionnaires.

To conclude, adolescents with ADHD seem to experience more disturbed subjective sleep parameters and sleep problems compared to their TD peers. This is particularly concerning, because disrupted sleep worsens ADHD symptom impairment (Becker, Epstein et al., 2019). Consequently, sleep should be assessed routinely in the diagnostic process of ADHD. The current meta‐analytic review also shows that there is a lack of research on sleep in adolescents with and without ADHD and hopefully spurs much needed research in this area. First, a major focus should be on the inclusion of objective measurements, such as actigraphy and polysomnography or other technologies such as commercial wearables or portable EEG applications to measure sleep in the home setting. Second, other factors possibly influencing sleep, such as medication or comorbidities, should be assessed and controlled for. Lastly, variables related to sleep hygiene need further attention. It has been shown that sleep hygiene does significantly impact the sleep of adolescents with ADHD (Martin et al., 2020) and it might be one of the main modifiable variables to improve sleep.

## AUTHOR CONTRIBUTIONS

**Finja Marten**: Conceptualization; Data curation; Formal analysis; Investigation; Methodology; Project administration; Resources; Software; Validation; Visualization; Writing – original draft; Writing – review & editing. **Lena Keuppens**: Conceptualization; Data curation; Formal analysis; Investigation; Methodology; Project administration; Resources; Software; Validation; Visualization; Writing – original draft; Writing – review & editing. **Dieter Baeyens**: Funding acquisition; Supervision; Writing – review & editing. **Bianca E. Boyer**: Supervision; Writing – review & editing. **Marina Danckaerts**: Funding acquisition; Writing – review & editing. **Samuele Cortese**: Writing – review & editing. **Saskia Van der Oord**: Funding acquisition; Supervision; Writing – review & editing.

## CONFLICT OF INTEREST STATEMENT

MD is participating in a Takeda‐sponsored clinical trial in ADHD. SC serves on the JCPP Advances Editorial Advisory Board. SC declares honoraria and reimbursement for travel and accommodation expenses for lectures from the following non‐profit associations: Association for Child and Adolescent Central Health (ACAMH), Canadian ADHD Alliance Resource (CADDRA), British Association of Pharmacology (BAP), and from Healthcare Convention for educational activity on ADHD. The remaining authors have declared that they have no competing or potential conflicts of interest.

## ETHICAL CONSIDERATIONS

Ethical approval was not required for this review article.

## Supporting information

Supporting Information S1

## Data Availability

Data sharing is not applicable to this article as no new data were created or analyzed in this study.
