# Supplementary material for: Sleep parameters and problems in adolescents with and without ADHD: A systematic review and meta‐analysis
Source: JCPP Adv. 2023 Mar 11;3(3):e12151. doi: 10.1002/jcv2.12151 (PMC10501691; doi:10.1002/jcv2.12151)
Supplement: Supplementary file 1 — Supporting Information S1 [file JCV2-3-e12151-s001.docx]

**Supporting Information**

**Table S1**

*Newcastle Ottawa Scale assessment for each study*

**Bauermann (2010)**

| NOS Item | Star | Explanation |
| --- | --- | --- |
| SELECTION |  |  |
| 1. Is the case definition adequate? | * | “Respondents were categorized into four non-overlapping symptom group (inattentive, hyperactive/impulsive, combined ADHD group and non-ADHD controls) using the cut-off scores on the DSM-IV ADHD scales from the Conners Adult ADHD Rating Scale; CAARS scoring manual.” |
| 2. Representativeness of the cases |  | “Participants were attending a moderate sized university in central Ontario, Canada. The participants were recruited by their course instructor to participate “in a study on sleep, emotion and health” at the end of a regularly scheduled lecture.” |
| 3. Selection of controls | * | “Controls were also attending the same moderate sized university in central Ontario, Canada.” |
| 4. Definition of controls | * | “Respondents with standard scores (T-scores) of 50 or less on the CAARS were classified as non-ADHD controls.” |
| COMPARABILITY |  |  |
| 1. Comparability of cases and controls on the basis of the design or analysis | ** | Age- and gender-matched healthy controls. |
| EXPOSURE |  |  |
| 1. Ascertainment of Exposure |  | Sleep Problems Inventory. |
| 2. Same method of ascertainment for cases and controls | * | All participants filled out the SPI. |
| 3. Non-response rate |  | This study had a 90% response rate. |

Total number of stars: 6 (fair)

**Becker et al. (2021)**

| NOS Item | Star | Explanation |
| --- | --- | --- |
| SELECTION |  |  |
| 1. Is the case definition adequate? | * | “Based on a comprehensive evaluation, 58 of the adolescents were diagnosed with DSM-5 ADHD...” |
| 2. Representativeness of the cases |  | “Participants with and without ADHD were initially recruited in eighth grade from local schools across Ohio, Kentucky, and Virginia (United States).” |
| 3. Selection of controls | * | “Participants with and without ADHD were initially recruited in eighth grade from local schools across Ohio, Kentucky, and Virginia (United States).” |
| 4. Definition of controls | * | “Based on a comprehensive evaluation, … remaining participants (n = 64) comprising a comparison sample without ADHD” |
| COMPARABILITY |  |  |
| 1. Comparability of cases and controls on the basis of the design or analysis | ** | “Race, family income, medication status, and comorbid diagnosis status were retained as covariates in the primary analyses given significant bivariate associations with sleep problems during COVID-19. Participant sex, ethnicity, age, and social media use were not associated with any of the primary sleep outcomes and were therefore not included in the models.” |
| EXPOSURE |  |  |
| 1. Ascertainment of Exposure |  | Sleep Habits Survey and Sleep Disturbance Scale for Children |
| 2. Same method of ascertainment for cases and controls | * | All participants filled out the same questionnaires. |
| 3. Non-response rate |  | No information. |

Total number of stars: 6 (fair)

**Becker et al. (2019)**

| NOS Item | Star | Explanation |
| --- | --- | --- |
| SELECTION |  |  |
| 1. Is the case definition adequate? | * | “All participants underwent a comprehensive ADHD diagnostic evaluation in accordance with the Fifth edition of the Diagnostic and Statistical Manual for Mental Disorders (DSM-5) criteria and the Parent version of Children’s Interview for Psychiatric Syndromes. Specifically, participants were included in the ADHD group if parents reported ≥6 symptoms of inattention at clinically significant levels; presence of ADHD symptoms prior to age 12 years, presence of ADHD symptoms in two or more settings (e.g. home, school), evidence that symptoms contribute to home, academic, and/or social impairment; and symptoms of ADHD were not better explained by another mental disorder.” |
| 2. Representativeness of the cases |  | “Participants were in eight grade and were recruited from local schools across two sites in the Southeastern and Midwestern United States.” But it was not clear whether participants were representative of the school population. |
| 3. Selection of controls | * | “Controls were selected from the same schools.” |
| 4. Definition of controls | * | “Participants were included in the comparison group if the parent endorsed <4 symptoms of ADHD in both domains (i.e. inattention, hyperactivity/impulsivity) on the P‐ChIPS. Additionally, both parent and adolescent report on the P‐ChIPS and ChIPS were used to determine common mental health diagnoses (i.e. mood and anxiety disorders, disruptive behavior disorders, obsessive–compulsive disorder).” |
| COMPARABILITY |  |  |
| 1. Comparability of cases and controls on the basis of the design or analysis | ** | This study controlled for age, primary household income, pubertal development, race, medication use, externalizing diagnosis, anxiety diagnosis, depression diagnosis, and gender. |
| EXPOSURE |  |  |
| 1. Ascertainment of Exposure | * | Actigraphy, ActiGraph GT9X (secure record), was used to measure sleep. Moreover, a sleep diary, the Sleep Habits Survey, Sleep Disturbance Scale for Children and the Teacher’s Daytime Sleepiness Questionnaire were administered. |
| 2. Same method of ascertainment for cases and controls | * | All participants wore the actigraphs and filled out sleep diaries and all questionnaires. |
| 3. Non-response rate |  | No information. |

Total number of stars: 7 (good)

**Cadman et al. (2016)**

| NOS Item | Star | Explanation |
| --- | --- | --- |
| SELECTION |  |  |
| 1. Is the case definition adequate? | * | “Cases received a clinical diagnosis of combined type ADHD or hyperkinetic disorder and the clinical diagnosis of DSM-IV combined type had to be confirmed following a detailed research assessment. The baseline diagnosis of ADHD was made according to the Parent Account of Childhood Symptoms (PACS). A standardized algorithm for PACS was applied to all raw PACS data to yield diagnoses based on operational DSM-IV criteria for ADHD. Moreover, the Diagnostic Interview for ADHD in adults (DIVA) was used to assess ADHD symptoms at follow-up.” |
| 2. Representativeness of the cases |  | “Participants were recruited from the UK-London subset of the International Multi-Centre ADHD Genetics (IMAGE) Project; a family and molecular genetic study of ADHD with sample collection between June 2003 and January 2006. Families were recruited to the IMAGE study by referral from child and adolescent clinics in the southeast of England on the basis that they had received a clinical diagnosis of combined type ADHD or hyperkinetic disorder and had at least one surviving biological sibling aged 5–17 years of age.” |
| 3. Selection of controls | * | To compare mental health outcomes with population norms, comparison data was acquired from the Adult Psychiatric Morbidity Study 2007 (APMS; www.ic.nhs.uk/pubs/psychiatricmorbidity07). |
| 4. Definition of controls | * | “Controls did not meet criteria for ADHD.” |
| COMPARABILITY |  |  |
| 1. Comparability of cases and controls on the basis of the design or analysis | * | A gender-matched subset was used and the effect of various descriptive characteristics on the outcome variables was examined, (e.g. age, IQ, childhood medication status, comorbid ODD/CD, …). |
| EXPOSURE |  |  |
| 1. Ascertainment of Exposure |  | Clinical Interview Schedule-Revised (CIS-R) |
| 2. Same method of ascertainment for cases and controls | * | All participants did the CIS-R. |
| 3. Non-response rate |  | This study had a response rate of 57.8% at follow up. |

Total number of stars: 5 (fair)

**Frick et al. (2022)**

| NOS Item | Star | Explanation |
| --- | --- | --- |
| SELECTION |  |  |
| 1. Is the case definition adequate? | * | “Diagnostic status, including ADHD presentation, was obtained from medical records (S1) or reported by parents  (S2 and S3). Additionally, in S1 all participants were interviewed with a structured diagnostic interview performed by clinical psychologists (MINI-kid).” “…due to these differing diagnostic methods and that some assessments were conducted in previous years, ADHD presentation was based on dichotomized cut-off points using the Adult ADHD Adolescent version, Parent-Report Scale, (ASRS-AP) as recommended by Kessler and validated in a Swedish population.” “…six or more symptoms (five or more for age 17 and older, in accordance with the DSM-5) from each symptom domain respectively (i.e., inattention and hyperactivity/ impulsivity) informed on ADHD presentation (i.e., ADHDC, ADHD-I, and ADHD-H).” |
| 2. Representativeness of the cases |  | Participants with ADHD were “…recruited via Child and Adolescent Psychiatric (CAP) units in Sweden (i.e. both rural and urban). |
| 3. Selection of controls |  | “The control sample (S3; aged 14–19 years) was recruited via local schools as a reference group.” |
| 4. Definition of controls | * | Less than six symptoms or less than five (for age 17 and older) on the ASRS-AP. |
| COMPARABILITY |  |  |
| 1. Comparability of cases and controls on the basis of the design or analysis | * | This study controlled for age. |
| EXPOSURE |  |  |
| 1. Ascertainment of Exposure |  | The adolescents reported on sleep difficulties using the Karolinska sleep questionnaire (KSQ). |
| 2. Same method of ascertainment for cases and controls | * | The same questionnaire was administered. |
| 3. Non-response rate |  | No information. |

Total number of stars: 4 (poor)

**Gregory et al. (2017)**

| NOS Item | Star | Explanation |
| --- | --- | --- |
| SELECTION |  |  |
| 1. Is the case definition adequate? | * | “Diagnosis at age 18 was ascertained based on private structured interviews with participants regarding 18 symptoms of inattention and hyperactivity-impulsivity according to DSM-5 criteria. In childhood the ADHD diagnosis was ascertained by mother and teacher reports according to the criteria of the DSM-IV.” |
| 2. Representativeness of the cases | * | “Participants were members of the Environmental Risk (ERisk) Longitudinal Twin Study, which tracks the development of a birth cohort of 2,232 British children. The sample was drawn from a larger birth register of twins born in England and Wales in 1994–1995.” |
| 3. Selection of controls | * | “Controls were also members of the ERisk Longitudinal Twin Study.” |
| 4. Definition of controls | * | “Controls did not meet criteria for an ADHD diagnosis based on the DSM-5 criteria.” |
| COMPARABILITY |  |  |
| 1. Comparability of cases and controls on the basis of the design or analysis | ** | This study controlled for gender, maternal insomnia, ADHD medication, whether they had young children and comorbid mental health problems. |
| EXPOSURE |  |  |
| 1. Ascertainment of Exposure |  | Pittsburgh Sleep Quality Index (PSQI) |
| 2. Same method of ascertainment for cases and controls | * | All participants filled out the PSQI. |
| 3. Non-response rate |  | This study had a response rate of 93%. |

Total number of stars: 7 (good)

**Hysing et al. (2016)**

| NOS Item | Star | Explanation |
| --- | --- | --- |
| SELECTION |  |  |
| 1. Is the case definition adequate? | * | “Symptoms of inattention and hyperactivity were measured using the official Norwegian translation of the Adult ADHD Self-Report Scale. Youth with a score above the 90^th^ percentile were defined as ASRS high-scorers.” |
| 2. Representativeness of the cases | * | “All adolescents from all around Norway born between 1993 and 1995 were invited to participate.” |
| 3. Selection of controls | * | “All adolescents from Norway born between 1993 and 1995 were invited to participate.” |
| 4. Definition of controls | * | “Youth with a score beneath the 90^th^ percentile were defined as ASRS low-scorers.” |
| COMPARABILITY |  |  |
| 1. Comparability of cases and controls on the basis of the design or analysis | ** | The study controlled for age, gender, depressive symptoms, use of ADHD medication and use of electronic devices. |
| EXPOSURE |  |  |
| 1. Ascertainment of Exposure |  | Self-reported bedtime and risetime, subjective sleep need, difficulties initiating and maintaining sleep was rated on a three-point Likert scale. Insomnia was operationalized in accordance with Lichtstein et al.’s quantitative criteria for insomnia and delayed sleep phase syndrome according to the ICSD-R criteria. |
| 2. Same method of ascertainment for cases and controls | * | All participants filled out all the questionnaires. |
| 3. Non-response rate |  | This study had a participation rate of 53%. |

Total number of stars: 7 (good)

**Liu et al. (2019)**

| NOS Item | Star | Explanation |
| --- | --- | --- |
| SELECTION |  |  |
| 1. Is the case definition adequate? | * | “The Youth Self-Report (YSR) of Achenbach Child Behavior Checklist (CBCL) was used to measure ADHD. The 90th percentile of the scale score at baseline survey of the entire cohort was used as the cutoff for clinically relevant ADHD symptoms.” |
| 2. Representativeness of the cases | * | “Participants were sampled from 5 middle and 3 high schools in 3 counties of Shandong, with consideration of the representativeness of adolescent students in the region, prior study collaboration, convenience, and budget for at least 3 waves of data collection.” |
| 3. Selection of controls | * | “Controls were also sampled from 5 middle and 3 high schools in 3 counties of Shandong, with consideration of the representativeness of adolescent students in the region, prior study collaboration, convenience, and budget for at least 3 waves of data collection.” |
| 4. Definition of controls | * | “Participants who scored lower than the 90^th^ percentile at the YSR of the CBCL, were assigned to the control group.” |
| COMPARABILITY |  |  |
| 1. Comparability of cases and controls on the basis of the design or analysis | ** | “Covariates were adolescent and family demographic variables that were significantly different between adolescents with and without clinically ADHD symptoms at baseline.” |
| EXPOSURE |  |  |
| 1. Ascertainment of Exposure |  | “The Adolescent Health Questionnaire (AHQ) was administered and four questions developed by the 2003 NIH Restless Legs Syndrome Diagnosis and Epidemiology workshop to assess RLS symptoms.” |
| 2. Same method of ascertainment for cases and controls | * | All participants filled out the AHQ and the four questions to assess RLS. |
| 3. Non-response rate |  | Follow-up rate of 82%. |

Total number of stars: 7 (good)

**Mullin et al. (2011)**

| NOS Item | Star | Explanation |
| --- | --- | --- |
| SELECTION |  |  |
| 1. Is the case definition adequate? | * | “Participants in the ADHD-C group met DSM-IV-TR criteria for this condition. The parent completed the Kiddie-Schedule for Affective Disorders and Schizophrenia-Present and Lifetime version (KSADS-PL).” |
| 2. Representativeness of the cases |  | “Participants were recruited from parent-support groups, mental health support listservs, postings at mental health clinics, and an online community message board in the San Francisco Bay Area, CA, USA. Participants with ADHD-C were typically referred from physicians’ offices and community postings.” |
| 3. Selection of controls | * | “Participants were recruited from parent-support groups, mental health support listservs, postings at mental health clinics, and an online community message board in the San Francisco Bay Area, CA, USA. Controls were typically referred from physicians’ offices and community postings.” |
| 4. Definition of controls | * | “Healthy controls had not received any clinical diagnoses or received treatment for psychological problems. The parent completed the Kiddie-Schedule for Affective Disorders and Schizophrenia-Present and Lifetime version (KSADS-PL).” |
| COMPARABILITY |  |  |
| 1. Comparability of cases and controls on the basis of the design or analysis | * | “Analysis of covariance (ANCOVA) was used to include sex as a covariate.” |
| EXPOSURE |  |  |
| 1. Ascertainment of Exposure | * | Participants were asked to complete four consecutive nights of sleep monitoring at home, wearing an actigraph, Actiwatch-64 (secure record), and keeping a sleep diary (written self-report). |
| 2. Same method of ascertainment for cases and controls | * | Both ADHD-C participants and healthy controls wore the actigraph and kept a sleep diary. |
| 3. Non-response rate |  | No information. |

Total number of stars: 6 (fair)

**Ng et al. (2017)**

| NOS Item | Star | Explanation |
| --- | --- | --- |
| SELECTION |  |  |
| 1. Is the case definition adequate? | * | “Servicemen with a diagnosis of ADHD were identified via  comprehensive electronic medical records. For a diagnosis of ADHD to be recorded into the  SAF electronic medical system, an official medical report from a certified psychiatrist detailing the diagnosis was required. Moreover, participants filled out the Adult ADHD Self-Report Scale, Version 1.1 (ASRS). If four or more marks appeared in pre-identified boxes within the questionnaire, the patient was considered to have symptoms highly consistent with ADHD.” |
| 2. Representativeness of the cases | * | “This is a part of a cross-sectional study, which collected data from participants who enlisted between 2008 and 2009. The aim of this study was to include an equal number of servicemen with and without ADHD. All servicemen with “Attention Deficit Disorder,” “Attention Deficit Hyperactivity Disorder,” or “Hyperkinesis” recorded in the SAF electronic medical system were included in this study.” |
| 3. Selection of controls | * | Controls were also service men. |
| 4. Definition of controls | * | “The medical records of controls were checked to ensure that they were not previously diagnosed with ADHD.” |
| COMPARABILITY |  |  |
| 1. Comparability of cases and controls on the basis of the design or analysis | ** | “Controls were matched for gender, age, nature of work and educational level.” |
| EXPOSURE |  |  |
| 1. Ascertainment of Exposure |  | Questions about sleep history (time needed to fall asleep, number of times awakened (M) time to sleep after interruption and early morning awakenings). |
| 2. Same method of ascertainment for cases and controls | * | All participants were asked questions about their sleep history. |
| 3. Non-response rate |  | No information about controls. |

Total number of stars: 7 (good)

**Takahashi et al. (2016)**

| NOS Item | Star | Explanation |
| --- | --- | --- |
| SELECTION |  |  |
| 1. Is the case definition adequate? | * | “Suspected ADHD was determined using the ADHD Self-Report Scale (ASRS)-v1.1 Part A, as previously described. Scoring a level of 4 items or more, of 6, was defined as suspected ADHD.” |
| 2. Representativeness of the cases |  | “A total of 375 female college students among 439 students, aged 19.2 ± 1.3 years, were enrolled in this cross-sectional study, and met the following criteria: (1) they voluntarily undertook the self-reported questionnaires at the women’s college, Matsuyama, Japan, from April to May 2015, (2) they provided written, informed consent (Table 1).” |
| 3. Selection of controls | * | Same women’s colleges |
| 4. Definition of controls | * | Controls have neither ADHD neither ASD. Soring a level of 3 items or less of 6, was defined as non-ADHD and scoring a level of 32 items or less of 50 was defined as non-ASD. |
| COMPARABILITY |  |  |
| 1. Comparability of cases and controls on the basis of the design or analysis |  | No covariates were included. |
| EXPOSURE |  |  |
| 1. Ascertainment of Exposure |  | National Health Nutritional Survey. |
| 2. Same method of ascertainment for cases and controls | * | All participants answered the same questions. |
| 3. Non-response rate |  | A total of 375 female college students among 439 students were enrolled in this cross-sectional study. |

Total number of stars: 4 stars (poor)

**Voinescu et al. (2012)**

| NOS Item | Star | Explanation |
| --- | --- | --- |
| SELECTION |  |  |
| 1. Is the case definition adequate? | * | “To determine ADHD, cut-off points on the Adult Self-Report Scales (ASRS) and the Barkley Adult ADHD Rating Scale-IV (BAARS-IV) Current Symptoms were used.” |
| 2. Representativeness of the cases |  | “Participants were students attending the under-graduate and master degree programs of the Faculty of Psychology at Faculty of Psychology at ‘‘Alexandru Ioan Cuza’’ University in Iasi and at ‘‘Babes-Bolyai’’ University in Cluj-Napoca, Romania. Collection took place during the first and second semesters of 2011.” But it was not clear whether participants were representative of the university population. |
| 3. Selection of controls | * | “Controls were also students attending the under-graduate and master degree programs of the Faculty of Psychology at Faculty of Psychology at ‘‘Alexandru Ioan Cuza’’ University in Iasi and at ‘‘Babes-Bolyai’’ University in Cluj-Napoca, Romania.” |
| 4. Definition of controls | * | “Cut-off points on the ASRS and the BAARS-IV determined the control group.” |
| COMPARABILITY |  |  |
| 1. Comparability of cases and controls on the basis of the design or analysis | ** | “…randomly matched the ADHD-likely participants by age and sex with 46 ‘healthy controls’” |
| EXPOSURE |  |  |
| 1. Ascertainment of Exposure |  | The Sleep Disorders Questionnaire (SDW), the Sleep Condition Indicator (SCI), Composite Scale of Morningness (CSM) and the Sleep Timing Questionnaire (STQ) were administered. |
| 2. Same method of ascertainment for cases and controls | * | All participants filled out the SDW, SCI, CSM and the STQ. |
| 3. Non-response rate |  | No information. |

Total number of stars: 6 (fair)

**Zerón‑Rugerio et al. (2021)**

| NOS Item | Star | Explanation |
| --- | --- | --- |
| SELECTION |  |  |
| 1. Is the case definition adequate? | * | ADHD RS-IV >= 1.5 SD |
| 2. Representativeness of the cases |  | “The cases were recruited in the ADHD Unit of the Department of Child and Adolescent Psychiatry and Psychology of the Hospital of Sant Joan de Déu (Barcelona, Spain).” |
| 3. Selection of controls | * | “Controls were recruited from the ADHD patients’ classmates (40%) and from patients attending in other hospital services (60%) (i.e. minor surgery ambulatory, or similar ones).” |
| 4. Definition of controls | * | ADHD RS-IV < 1.5 SD |
| COMPARABILITY |  |  |
| 1. Comparability of cases and controls on the basis of the design or analysis | ** | Gender and aged matched. |
| EXPOSURE |  |  |
| 1. Ascertainment of Exposure | * | Actigraph, Sleep Disturbance Scale for Children (SDSC), Munich Chronotype Questionnaire |
| 2. Same method of ascertainment for cases and controls | * | All participants wore an actigraph. |
| 3. Non-response rate |  | No info |

Total number of stars: 7 (good)

**Table S2**

*Information on Excluded Studies*

|  | Study | Reason for exclusion |
| --- | --- | --- |
|  | Adler et al. (2019) | Participants are too old |
|  | Akinci et al. (2015) | Participants are too young |
|  | Arns et al. (2014) | Participants are too old |
|  | Ashou Helal (2013) | Poster abstract, no full text |
|  | Azadi and Ebrahimi (2015) | Article in Iranian |
|  | Bader et al. (2003) | Poster abstract, no full text |
|  | Ball et al. (1997) | Participants are too young |
|  | Bauermann (2011) | Poster abstract, no full text |
|  | Bauermann and Majeski (2003) | Poster abstract, no full text |
|  | Becker et al. (2018) | ADHD as continuous construct |
|  | Becker et al. (2014) | ADHD as continuous construct |
|  | Bessey et al. (2013) | Participants are too young |
|  | Bestmann et al. (2019) | Participants are too young |
|  | Bijlenga et al. (2013) | Participants are too old |
|  | Bilgin et al. (2018) | Wrong direction of the effect |
|  | Bioulac et al. (2015) | Participants are too old |
|  | Bioulac et al. (2016) | Poster abstract, no full text |
|  | Bioulac et al. (2021) | Participants are too old |
|  | Bioulac et al. (2014) | Poster abstract and participants are too young |
|  | Bjorvatn et al. (2017) | Participants are too old |
|  | Bjorvatn et al. (2017) | Poster abstract, no full text |
|  | Bogdan and Reeves (2018) | Participants are too old |
|  | Bolden et al. (2019) | ADHD as continuous construct |
|  | Boonstra et al. (2007) | Participants are too old |
|  | Bourchtein et al. (2019) | Same sample as Becker et al. (2019b) |
|  | Brevik et al. (2016) | Poster abstract, no full text |
|  | Bruni et al. (2021) | No verification of ADHD |
|  | Budagova, and Bahadir (2019) | Poster abstract, no full text |
|  | Bumb et al. (2016) | Participants are too old |
|  | Campos et al. (2003) | Article in Spanish |
|  | Cao et al. (2012) | Article in Chinese |
|  | Chang et al. (2018) | No differentiation between ADHD and learning difficulties |
|  | Chiang et al. (2010) | Participants are too young |
|  | Chin et al. (2018) | Participants are too young |
|  | Chu et al. (2020) | Participants are too young |
|  | Cifre et al. (2019) | Poster abstract, no full text |
|  | Cohen et al. (2020) | Participants are too old |
|  | Coogan et al. (2019) | Participants are too old |
|  | Corkum et al. (2016) | No appropriate comparison group; waitlist control with ADHD |
|  | Costanzo et al. (2016) | Poster abstract, no full text |
|  | Cremone-Caira et al. (2020) | Participants are too young |
|  | Cruz et al. (2007) | Poster abstract, no full text |
|  | Cusick et al. (2018) | Same sample as Becker et al. (2019b) |
|  | Cusick et al. (2020) | Same sample as Becker et al. (2019b) |
|  | Dagan et al. (1997) | Participants are too young |
|  | Darchia et al. (2019) | Poster abstract, no full text |
|  | Darchia et al. (2019) | Poster abstract, no full text |
|  | Day and Abmayr (1998) | Participants are too young |
|  | De Dea et al. (2018) | Participants are too young |
|  | Desrochers et al. (2015) | Poster abstract, no full text |
|  | Dimakos et al. (2019) | Poster abstract, no full text |
|  | Dimakos et al. (2019) | Poster abstract, no full text |
|  | Engelhardt et al. (2013) | Participants are too young |
|  | Estrada-Prat et al. (2019) | Participants are too young |
|  | Evren et al. (2019) | No Mean and SD of the effects and wrong direction of the effect |
|  | Eyuboglu and Eyuboglu (2018) | Participants are too young |
|  | Faedda et al. (2016) | Participants are too young |
|  | Fallone et al. (2005) | Poster abstract, no full text |
|  | Faraone et al. (2019) | Participants are too young and no verification of ADHD |
|  | Felt et al. (2010) | Poster abstract, no full text |
|  | Ferri et al. (2013) | Participants are too young |
|  | Fisher et al. (2014) | No appropriate comparison group; ADHD and ADHDplus (comorbid disorder) |
|  | Frye et al. (2017) | Poster abstract, no full text |
|  | Frye et al. (2018) | No verification of ADHD |
|  | Fuller-Thomson et al. (2016) | Participants are too old |
|  | Furrer et al. (2019) | Participants are too young |
|  | Gamble et al. (2013) | Participants are too old |
|  | Garbazza et al. (2018) | Participants are too old |
|  | Gau (2006) | Participants are too young |
|  | Gau and Chiang (2009) | Participants are too young |
|  | Gau et al. (2007) | No verification of ADHD |
|  | Gau et al. (2010) | Participants are too young |
|  | Gaultney (2014) | No verification of ADHD |
|  | Gaultney et al. (2019) | Full text not available |
|  | Gaultney et al. (2005) | Participants are too young |
|  | Greenhill et al. (1983) | Participants are too young |
|  | Gruber et al. (2000) | Participants are too young |
|  | Hansen et al. (2013) | Participants are too young |
|  | Harsh et al. (2001) | Poster abstract, no full text |
|  | Hazari et al. (2015) | Participants are too young |
|  | Helfer and Asherson (2019) | Poster abstract, no full text |
|  | Helfer et al. (2020) | Participants are too old |
|  | Helwig (2011) | Participants are too young |
|  | Hong et al. (2020) | Participants are too young |
|  | Huang et al. (2006) | Participants are too young |
|  | Hysing et al. (2020) | Same sample as Hysing et al. (2016) |
|  | Ibilola and Silva (2017) | Poster abstract, no full text |
|  | Ito et al. (2017) | Poster abstract, no full text |
|  | Ito et al. (2017) | Poster abstract, no full text |
|  | Ito and Narisawa (2019) | Poster abstract, no full text |
|  | Jackson and Vaughn (2017) | Participants are too young |
|  | Janusis (2011) | Only ADHD symptoms |
|  | Johnson & Suhr (2021) | No verification of ADHD |
|  | Kalil Neto and Nunes (2016) | Participants are too young |
|  | Karadag et al. (2019) | Participants are too young |
|  | Khan (1982) | Participants are too young |
|  | Khurshid and Spire (2006) | Poster abstract, no full text |
|  | Kidd (2009) | Participants are too young |
|  | Kirov et al. (2017) | Participants are too young |
|  | Kirov et al. (2007) | Participants are too young |
|  | Kirov et al. (2004) | Participants are too young |
|  | Kirov et al. (2011) | Poster abstract, no full text |
|  | Kirov et al. (2013) | Poster abstract, no full text |
|  | Konofal et al. (2007) | Participants are too young |
|  | Kooij et al. (2001) | Participants are too old |
|  | Kortesoja et al. (2020) | No verification of ADHD |
|  | Kwon et al. (2020) | ADHD as continuous construct |
|  | Kwon et al. (2020) | ADHD as continuous construct |
|  | Lam and Yang (2008) | ADHD as continuous construct |
|  | Langberg et al. (2019) | Same sample as Becker et al. (2019) |
|  | Li et al. (2009) | Participants are too young |
|  | Lim et al. (2008) | Participants are too young |
|  | Lufi & Tzischinski (2014) | No verification of ADHD |
|  | Lunsford-Avery et al. (2018) | Poster abstract, no full text |
|  | Lunsford-Avery et al. (2019) | Poster abstract, no full text |
|  | Lundervold et al. (2019) | Poster abstract, no full text |
|  | Lundervold et al. (2020) | Participants are too old |
|  | Madiouni et al. (2020) | Participants are too old |
|  | Marta et al. (2010) | Poster abstract, no full text |
|  | Mayes et al. (2009) | Participants are too young and inappropriate study design |
|  | Mayes et al. (2009) | Participants are too young |
|  | Mayes et al. (2021) | No control group |
|  | McGowan and Coogan (2016) | Poster abstract, no full text |
|  | McGowan et al. (2016) | Participants are too old |
|  | Merikanto et al. (2019) | No mean and standard deviation |
|  | Merino-Andreu et al. (2008) | Poster abstract, no full text |
|  | Mick et al. (2000) | Participants are too young |
|  | Migliarese et al. (2020) | Participants aretoo old |
|  | Miniksar & Özdemir (2021) | Participants are too young |
|  | Montagni et al. (2019) | No verification of ADHD |
|  | Moreau et al. (2014) | Participants are too young |
|  | Nakatani et al. (2013) | Participants are too young |
|  | Navarro-Soria et al. (2021) | Measurement solely during confinement due to COVID-19 |
|  | Niers et al. (2006) | Poster abstract, no full text |
|  | O’Brien et al. (2003) | Participants are too young |
|  | O’Brien et al. (2003) | Participants are too young |
|  | O’Brien and Mindell (2006) | Poster abstract, no full text |
|  | Ogundele (2018) | Poster abstract, no full text |
|  | Owens et al. (2008) | Participants are too young |
|  | Park et al. (2011) | Participants are too old |
|  | Philip et al. (2015) | Poster abstract, no full text |
|  | Philip et al. (2015) | Participants are too old |
|  | Philipsen et al. (2005) | Participants are too old |
|  | Picchietti et al. (1999) | Participants are too young |
|  | Poirier and Corkum (2018) | Participants are too young |
|  | Prehn-Kristensen et al. (2011) | Participants are too young |
|  | Reimer et al. (2007) | Participants are too old |
|  | Ricci et al. (2022) | No association ADHD and sleep |
|  | Ringli et al. (2013) | Participants are too young |
|  | Rogers et al. (2017) | Participants are too old |
|  | Roy et al. (2018) | Participants are too old |
|  | Saito et al. (2019) | Participants are too young |
|  | Saletin et al. (2017) | Participants are too young |
|  | San Mauro Martín et al. (2018) | Participants are too young |
|  | Sawyer et al. (2008) | Participants are too young |
|  | Schlarb and Gruenwald (2018) | Poster abstract, no full text |
|  | Schredl et al. (2007) | Participants are too old |
|  | Schredl et al. (2017) | Participants are too old |
|  | Shen et al. (2020) | Participants are too young |
|  | Sivertsen et al. (2015) | No appropriate comparison group |
|  | Sobanski et al. (2016) | Participants are too old |
|  | Sobanski et al. (2008) | Participants are too old |
|  | Spera et al. (2020) | Participants are too old |
|  | Stein (1999) | Participants are too young |
|  | Stein et al. (2002)] | Participants are too young |
|  | Stephens (2001) | Participants are too young |
|  | Stephens et al. (2013) | Participants are too young |
|  | Surman et al. (2009) | Participants are too old |
|  | Taillard et al. (2017) | Poster abstract, no full text |
|  | Thoma et al. (2020) | Participants are too young |
|  | Tidwell et al. (2016) | Poster abstract, no full text |
|  | Tomás et al. (2008) | Article in Spanish |
|  | Tonetti et al. (2017) | Participants are too old |
|  | Tonetti et al. (2018) | Participants are too old |
|  | Tsai et al. (2012) | Participants are too young |
|  | Tsai et al. (2017) | Participants are too young |
|  | Tsuji and Takeda (2018) | Conference paper, no full text |
|  | Vaidyanathan et al. (2016) | Participants are too young |
|  | Van Andel et al. (2020) | Participants are too old |
|  | Van Veen et al. (2010) | Participants are too old |
|  | Vélez-Galarraga et al. (2016) | Participants are too young |
|  | Virring et al. (2017) | Participants are too young |
|  | Vogel et al. (2016) | Poster abstract, no full text |
|  | Vogel et al. (2015) | Participants are too old and comorbid obesity in both ADHD and control group |
|  | Voinescu et al. (2011) | Poster abstract, no full text |
|  | Walker (2017) | Participants are too young |
|  | Waxmonsky et al. (2017) | Participants are too young |
|  | Weibel et al. (2017) | Participants are too old |
|  | Weiner et al. (2018) | Poster abstract, no full text |
|  | Wynchank et al. (2016) | Participants are too old |
|  | Wynchank et al. (2018) | Participants are too old |
|  | Yang et al. (2011) | Participants are too young |
|  | Yemula et al. (2019) | Poster abstract, no full text |
|  | Yeom et al. (2020) | No mean and standard deviation |
|  | Zarei et al. (2016) | Poster abstract, no full text |
|  | Zhao et al. (2021) | No association ADHD and sleep |

**Supplementary References**

1. Adler, L. A., Faraone, S. V., Sarocco, P., Atkins, N., & Khachatryan, A. (2019). Establishing US norms for the Adult ADHD Self‐Report Scale (ASRS‐v1. 1) and characterising symptom burden among adults with self‐reported ADHD. *International Journal of Clinical Practice, 73*(1), e13260. https://doi.org/10.1111/ijcp.13260
2. Akinci, G., Oztura, I., Hiz, S., Akdogan, O., Karaarslan, D., Ozek, H., & Akay, A. (2015). Sleep structure in children with attention-deficit/hyperactivity disorder. *Journal of Child Neurology, 30*(11), 1520-1525. https://doi.org/10.1177/0883073815573318
3. Arns, M., Feddema, I., & Kenemans, J. L. (2014). Differential effects of theta/beta and SMR neurofeedback in ADHD on sleep onset latency. *Frontiers in Human Neuroscience, 8*(1019), 1-10. https://doi.org/10.3389/fnhum.2014.01019
4. Ashou Helal, S. (2013, September 28-October 2*). Sleep disturbances in children with attention deficit* [Conference abstract]. 5^th^ World Congress on Sleep Medicine, Valencia, Spain. https://www.sciencedirect.com/journal/sleep-medicine/vol/14/suppl/S1
5. Azadi, M., & Ebrahimi, G. S. (2015, June 6-10). *Attention deficit/hyperactivity disorder and sleep problems: Mediating role of emotion regulation* [Conference abstract]. 29th Annual Meeting of Associated Professional Sleep Societies, LLC (APSS), Denver, CO, United States. https://sleepmeeting.org/wp-content/uploads/2018/10/abstractbook2015.pdf
6. Bader, G., Gillberg, C., Johnson, M., Kadesjo, B., & Rasmussen, P. (2003, June 3-8). *Activity and sleep in children with ADHD* [Conference abstract]. Annual Meeting of the Associated Professional Sleep Societies, LLC (APSS), Chicago, IL, United States. https://sleepmeeting.org/wp-content/uploads/2018/10/abstractbook2003.pdf
7. Ball, J. D., Tiernan, M., Janusz, J., & Furr, A. (1997). Sleep patterns among children with attention-deficit hyperactivity disorder: A reexamination of parent perceptions. *Journal of Pediatric Psychology, 22*(3), 389-398. https://doi.org/10.1093/jpepsy/22.3.389
8. Bauermann, T. (2011, September 10-14). *Sleep problems in young adults with attention-deficit-hyperactivity disorder (ADHD) symptomatology* [Conference abstract]. 4th International Congress of the Association of Sleep Medicine and 5th Conference of the Canadian Sleep Society, Quebec City, Canada. https://www.sciencedirect.com/journal/sleep-medicine/vol/12/suppl/S1?page=2
9. Bauermann, T., & Majeski, S. (2003, June 3-8). *ADHD and sleep problems in adults* [Conference abstract]. Annual Meeting of the Associated Professional Sleep Societies, LLC (APSS), Chicago, IL, United States. https://sleepmeeting.org/wp-content/uploads/2018/10/abstractbook2003.pdf
10. Becker, S. P., Jarrett, M. A., Luebbe, A. M., Garner, A. A., Burns, G. L., & Kofler, M. J. (2018). Sleep in a large, multi-university sample of college students: Sleep problem prevalence, sex differences, and mental health correlates. *Sleep Health, 4*(2), 174-181. https://doi.org/10.1016/j.sleh.2018.01.001
11. Becker, S. P., Luebbe, A. M., & Langberg, J. M. (2014). Attention-deficit/hyperactivity disorder dimensions and sluggish cognitive tempo symptoms in relation to college students’ sleep functioning. *Child Psychiatry and Human Development, 45*(6), 675-685. https://doi.org/10.1007/s10578-014-0436-8
12. Bessey, M., Richards, J., & Corkum, P. (2013). Sleep lab adaptation in children with attention-deficit/hyperactivity disorder and typically developing children. *Sleep disorders, 2013*, 1-4. https://doi.org/10.1155/2013/698957
13. Bestmann, A., Conzelmann, A., Baving, L., & Prehn-Kristensen, A. (2019). Associations between cognitive performance and sigma power during sleep in children with attention-deficit/hyperactivity disorder, healthy children, and healthy adults. *PloS One, 14*(10), 1-17. https://doi.org/10.1371/journal.pone.0224166
14. Bijlenga, D., Van der Heijden, K. B., Breuk, M., Van Someren, E. J. W., Lie, M. E., Boonstra, A. M., Swaab, H. J. T., & Kooij, J. J. S. (2013). Associations between sleep characteristics, seasonal depressive symptoms, lifestyle, and ADHD symptoms in adults*. Journal of Attention Disorders, 17*(3), 261-275. https://doi.org/10.1177/1087054711428965
15. Bilgin, A., Baumann, N., Jaekel, J., Breeman, L. D., Bartmann, P., Bäuml, J. G., Avram, M., Sorg, C. & Wolke, D. (2020). Early crying, sleeping, and feeding problems and trajectories of attention problems from childhood to adulthood. *Child Development, 91*(1), e77-e91. https://doi.org/10.1111/cdev.13155
16. Bioulac, S., Chaufton, C., Taillard, J., Claret, A., Sagaspe, P., Fabrigoule, C., Bouvard, M. P., & Philip, P. (2015). Excessive daytime sleepiness in adult patients with ADHD as measured by the Maintenance of Wakefulness Test, an electrophysiologic measure. *The Journal of Clinical Psychiatry, 76*(7), 943-948. https://doi.org/10.4088/JCP.14m09087
17. Bioulac, S., Sagaspe, P., Micoulaud-Franchi, J.-A., Altena, E., Taillard, J., Fabrigoule, C., & Philip, P. (2016, September 13-16). *Driving risks and attention deficit hyperactivity disorder don't forget to explore sleepiness* [Conference abstract]. 23rd Congress of the European Sleep Research Society, Bologna, Italy. https://onlinelibrary.wiley.com/doi/epdf/10.1111/jsr.12446
18. Bioulac, S., Sagaspe, P., Tron, E., Benard, A., Berthomier, C., Brandewinder, M., Philip, P., & Taillard, J. (2021). Does homeostatic sleep pressure buildup explain objective excessive daytime sleepiness in adults with ADHD? An exploratory study. *Frontiers in Psychiatry, 12*, 1-7. https://doi.org/10.3389/fpsyt.2021.586528
19. Bioulac, S., Tailard, J., Quéra-Salva, M. A., Sagaspe, P., & Philip, P. (2014, September 16-20). *Attention deficit hyperactivity symptoms, sleepiness and near-misses driving accidents among a population of French highway drivers* [Conference abstract]. 22nd Congress of the European Sleep Research Society, Tallinn, Estonia. https://onlinelibrary.wiley.com/doi/epdf/10.1111/jsr.12213
20. Bjorvatn, B., Brevik, E. J., Lundervold, A. J., Halmøy, A., Posserud, M.-B., Instanes, J. T., & Haavik, J. (2017). Adults with attention deficit hyperactivity disorder report high symptom levels of troubled sleep, restless legs, and cataplexy. *Frontiers in Psychology, 8*(1621), 1-11. https://doi.org/10.3389/fpsyg.2017.01621
21. Bjorvatn, B., Brevik, E. J., Lundervold, A. J., Halmøy, A., Posserud, M.-B., Instanes, J. T., & Haavik, J. (2017, December). *Troubled sleep, restless legs and cataplexy in adults with ADHD* [Conference abstract]. World Association of Sleep Medicine, Prague, Czech Republic. https://www.sciencedirect.com/journal/sleep-medicine/vol/40/suppl/S1
22. Bogdan, A. R., & Reeves, K. W. (2018). Sleep duration in relation to attention deficit hyperactivity disorder in American adults. *Behavioral Sleep Medicine, 16*(3), 235-243. https://doi.org/10.1080/15402002.2016.1188391
23. Bolden, J., Gilmore-Kern, J. E., & Fillauer, J. P. (2019). Associations among sleep problems, executive dysfunctions, and attention-deficit/hyperactivity disorder symptom domains in college students*. Journal of American College Health, 67*(4), 320-327. https://doi.org/10.1080/07448481.2018.1481070
24. Boonstra, A. M., Kooij, J. J. S., Oosterlaan, J., Sergeant, J. A., Buitelaar, J. K., & Van Someren, E. J. W. (2007). Hyperactive night and day? Actigraphy studies in adult ADHD: A baseline comparison and the effect of methylphenidate. *Sleep, 30*(4), 433-442. https://doi.org/10.1093/sleep/30.4.433
25. Bourchtein, E., Langberg, J. M., Cusick, C. N., Breaux, R. P., Smith, Z. R., & Becker, S. P. (2019). Technology use and sleep in adolescents with and without attention-deficit/hyperactivity disorder. *Journal of Pediatric Psychology*, 44(5), 517-526. https://doi.org/10.1093/jpepsy/jsy101
26. Brevik, E. J., Lundervold, A. J., Halmoy, A., Posserud, M.-B., Instanes, J. T., Bjorvatn, B., & Haavik, J. (2016, September 13-16). *Insomnia in adults with attention-deficit/hyperactivity disorder (ADHD): Impact of clinical subtype and pharmacological treatment* [Conference abstract]. 23rd Congress of the European Sleep Research Society, Bologna, Italy. https://onlinelibrary.wiley.com/doi/epdf/10.1111/jsr.
27. Bruni, O., Breda, M., Ferri, R., & Melegari, M. G. (2021). Changes in sleep patterns and disorders in children and adolescents with attention deficit hyperactivity disorders and autism spectrum disorders during the COVID-19 lockdown. *Brain Sciences, 11*(9), 1-12. https://doi.org/10.3390/brainsci11091139
28. Budagova, G., & Bahadir, A. T. (2019,. October 14-19). *Relationship between chronotype, sleep, and screen exposure in adolescents with ADHD* [Conference Abstract]. 66^th^ Annual Meeting of the American Academy of Child and Adolescent Psychiatry. https://jaacap.org/issue/S0890-8567(19)X0002-7
29. Bumb, J. M., Mier, D., Noelte, I., Schredl, M., Kirsch, P., Hennig, O., Liebrich, L., Fenske, S., Alm, B., Sauer, C., Leweke, F. M., & Sobanski, E. (2016). Associations of pineal volume, chronotype and symptom severity in adults with attention deficit hyperactivity disorder and healthy controls. *European Neuropsychopharmacology, 26*(7), 1119-1126. https://doi.org/10.1016/j.euroneuro.2016.03.016
30. Campos, C., Fernández, P., Mobarec, S., Claro, S., & Sánchez, I. (2003). The relationship between obstructive sleep apnea syndrome and attention deficit hyperactivity disorder: A study in Chilean schoolchildren. *Revista Chilena de Pediatria, 74*(1), 46-52. http://dx.doi.org/10.4067/S0370-41062003000100006
31. Cao, Y. L., Cui, Q. T., Tang, C. H., & Chang, X. (2012). Association of CLOCK gene T3111C polymorphism with attention deficit hyperactivity disorder and related sleep disturbances in children. *Chinese Journal of Contemporary Pediatrics, 14*(4), 285-288.
32. Chang, V. C., Chaput, J.-P., Roberts, K. C., Jayaraman, G., & Do, M. T. (2018). Factors associated with sleep duration across life stages: Results from the Canadian Health Measures Survey. *Health Promotion and Chronic Disease Prevention in Canada, 38*(11), 404-418. https://doi.org/10.24095/hpcdp.38.11.02
33. Chiang, H.‐L., Gau, S. S.-F., Ni, H.‐C., Chiu, Y.‐N., Shang, C.‐Y., Wu, Y.‐Y., Lin, L.-Y., Tai, Y.-M., & Soong, W.‐T. (2010). Association between symptoms and subtypes of attention‐deficit hyperactivity disorder and sleep problems/disorders. *Journal of Sleep Research, 19*(4), 535-545. https://doi.org/10.1111/j.1365-2869.2010.00832.x
34. Chin, W.-C., Huang, Y.-S., Chou, Y.-H., Wang, C.-H., Chen, K.-T., Hsu, J. F., & Hsu, S.-C. (2018). Subjective and objective assessments of sleep problems in children with attention deficit/hyperactivity disorder and the effects of methylphenidate treatment. *Biomedical Journal, 41*(6), 356-363. https://doi.org/10.1016/j.bj.2018.10.004
35. Chu, K.-C., Lu, H.-K., Huang, M.-C., Lin, S.-J., Liu, W.-I., Huang, Y.-S., Hsu, J.-F. & Wang, C.-H. (2020). Using mobile electroencephalography and actigraphy to diagnose attention-deficit/hyperactivity disorder: Case-control comparison study. *JMIR Mental Health, 7*(6), 1-10. http://doi.org/10.2196/12158
36. Cifre, A., Walters, K., & Budnick, C. (2019, September 20-25). *Do ADHD symptoms and insomnia interact to predict impaired executive functioning?* [Congress abstract]. 15th World Sleep congress, Vancouver, Canada. https://www.sciencedirect.com/journal/sleep-medicine/vol/64/suppl/S1
37. Cohen, A., Dan, O., Asraf, K., & Haimov, I. (2020). The sleepiness curve of young men with and without attention-deficit hyperactivity disorder (ADHD). *Behavioral Sleep Medicine, 18*(3), 321-333. https://doi.org/10.1080/15402002.2019.1583564
38. Coogan, A. N., Schenk, M., Palm, D., Uzoni, A., Grube, J., Tsang, A. H., Kolbe, I., McGowan, N. M., Wandschneider, R., Colla, M., Oster, H., Thome, J., & Faltraco, F. (2019). Impact of adult attention deficit hyperactivity disorder and medication status on sleep/wake behavior and molecular circadian rhythms. *Neuropsychopharmacology, 44*(7), 1198-1206. https://doi.org/10.1038/s41386-019-0327-6
39. Corkum, P., Lingley-Pottie, P., Davidson, F., McGrath, P., Chambers, C. T., Mullane, J., Laredo, S., Woodford, K., & Weiss, S. K. (2016). Better nights/better days-distance intervention for insomnia in school-aged children with/without ADHD: A randomized controlled trial. *Journal of Pediatric Psychology, 41*(6), 701-713. https://doi.org/10.1093/jpepsy/jsw031
40. Costanzo, A., Maisto, M., Arpino, M., Bruno, T., Cirace, F., Gazzillo, D., Vitolo, V., Ziviello, A., & Barbato, G. (2016, October 20-22). *Attention deficit/hyperactivity disorder symptoms and sleep quality assessed on healthy adults* [Conference abstract]. 28th ASM of Australasian Sleep Association and Australasian Sleep Technologist Association. https://onlinelibrary.wiley.com/doi/epdf/10.1111/jsr.12453
41. Cremone-Caira, A., Root, H., Harvey, E. A., McDermott, J. M., & Spencer, R. M. C. (2020). Effects of sleep extension on inhibitory control in children with ADHD: A pilot study*. Journal of Attention Disorders, 24(*4), 601-610. https://doi.org/10.1177/1087054719851575
42. Cruz, M., Valencia, I., Adams, R., Hardison, H., Marks, H., Khurana, D., Legido, A., & Kothare, S. (2007, June 9-14). *Polysomnographic abnormalities in patients with ADHD with diverse sleep problems* [Conference abstract]. 21st Annual Meeting of the Associated Professional Sleep Societies, LLC (APSS), Minneapolis, MN. https://sleepmeeting.org/wp-content/uploads/2018/10/abstractbook2007.pdf
43. Cusick, C. N., Isaacson, P. A., Langberg, J. M., & Becker, S. P. (2018). Last night's sleep in relation to academic achievement and neurocognitive testing performance in adolescents with and without ADHD. *Sleep Medicine, 52*, 75-79. https://doi.org/10.1016/j.sleep.2018.07.014
44. Cusick, C. N., Langberg, J. M., Breaux, R., Green, C. D., & Becker, S. P. (2020). Caffeine use and associations with sleep in adolescents with and without ADHD. *Journal of Pediatric Psychology, 45*(6), 643-653. https://doi.org/10.1093/jpepsy/jsaa033
45. Dagan, Y., Zeevi‐Luria, S., Sever, Y., Hallis, D., Yovel, I., Sadeh, A., & Dolev, E. (1997). Sleep quality in children with attention deficit hyperactivity disorder: An actigraphic study. *Psychiatry and Clinical Neurosciences, 51*(6), 383-386.
46. Darchia, N., Basishvili, T., Eliozishvili, M., Oniani, N., Tchintcharauli, T., Sakhelashvili, I., Oniani, T. J., Campbell, I. G., & Feinberg, I. (2019, September 21-25). *Sleep EEG as an index of brain maturation in typically developing and drug-naïve ADHD children* [Conference abstract]. 10th World Congress of Neuroscience, Daegu, Korea. https://www.sciencedirect.com/journal/ibro-reports/vol/6/suppl/S
47. Darchia, N., Tchintcharauli, T., Basishvili, T., Eliozishvili, M., Oniani, N., Oniani, T. J., Campbell, I. G., & Feinberg, I. (2019, September 7-10). *Sleep and cognitive functions in typically developing and drug-naïve ADHD children* [Conference abstract]. 32nd ECN Congress, Copenhagen, Denmark. https://www.sciencedirect.com/journal/european-neuropsychopharmacology/vol/29/suppl/S6?page=1
48. Day, H. D., & Abmayr, S. B. (1998). Parent reports of sleep disturbances in stimulant‐medicated children with attention‐deficit hyperactivity disorder. *Journal of Clinical Psychology, 54*(5), 701-716. https://doi.org/10.1002/(SICI)1097-4679(199808)54:5<701::AID-JCLP16>3.0.CO2-H
49. De Dea, F., Zanus, C., Carrozzi, M., Stecca, M., & Accardo, A. (2018, July 18-21*). Characteristics of EEG power spectrum during sleep spindle events in ADHD children* [Conference abstract]. 40th Annual International Conference of the IEEE Engineering in Medicine and Biology Society (EMBC), Honolulu, HI, United States. https://ieeexplore.ieee.org/xpl/conhome/8471725/proceeding
50. Desrochers, P., Cremone, A., Peterson, B., & Spencer, R. (2015, June 6-10). *Novel actigraphic measures demonstrate greater sleep disturbance in young children with symptoms of ADHD* [Conference Abstract]. 29th Annual Meeting of the Associated Professional Sleep Societies, LLC (APSS), Seattle, WA, United States. https://sleepmeeting.org/wp-content/uploads/2018/10/abstractbook2015.pdf
51. Dimakos, J., Somerville, G., Finn, C., Boursier, J., Keskinel, D., & Gruber, R. (2019, September 20-25). *Gender differences in sleep hygiene associated with poor sleep in adolescents with ADHD symptoms* [Conference abstract]*.* 15th World Sleep Congress, Vancouver, Canada. https://www.sciencedirect.com/journal/sleep-medicine/vol/64/suppl/S1
52. Dimakos, J., Somerville, G., Finn, C., Keskinel, D., Ionescu, A., Toro, C., Musacchio, S., Gruber, R., & Boursier, J. (2019, June 6-8). *Sleep behaviours and disturbances characterizing adolescents with ADHD symptoms* [Conference abstract]. 96th Annual Conference of the Canadian Pediatric Society, Toronto, Canada. https://academic.oup.com/pch/issue/24/Supplement_2
53. Engelhardt, C. R., Mazurek, M. O., & Sohl, K. (2013). Media use and sleep among boys with autism spectrum disorder, ADHD, or typical development. *Pediatrics, 132*(6), 1081-1089. https://doi.org/10.1542/peds.2013-2066
54. Estrada-Prat, X., Álvarez-Guerrico, I., Batlle-Vila, S., Camprodon-Rosanas, E., Martín-López, L. M., Álvarez, E., Romero, S., Elices, M., & Pérez, V. (2019). Sleep alterations in pediatric bipolar disorder versus attention deficit disorder. *Psychiatry Research, 275*, 39-45. https://doi.org/10.1016/j.psychres.2019.01.108
55. Evren, B., Evren, C., Dalbudak, E., Topcu, M., & Kutlu, N. (2019). The impact of depression, anxiety, neuroticism, and severity of internet addiction symptoms on the relationship between probable ADHD and severity of insomnia among young adults. *Psychiatry Research, 271*, 726-731. https://doi.org/10.1016/j.psychres.2018.12.010
56. Eyuboglu, M., & Eyuboglu, D. (2018). Behavioural sleep problems in previously untreated children with attention deficit hyperactivity disorder. *Psychiatry and Clinical Psychopharmacology, 28*(1), 19-24. https://doi.org/10.1080/24750573.2017.1368365
57. Faedda, G., Ohashi, K., Hernandez, M., McGreenery, C., Grant, M., Baroni, A., Polcari, A., & Teicher, M. (2016). Actigraph measures discriminate pediatric bipolar disorder from attention-deficit/hyperactivity disorder and typically developing controls. *Journal of Child Psychology and Psychiatry, 57*(6), 706-716. https://doi.org/10.1111/jcpp.12520
58. Fallone, G., Van Reen, E., Kelleher, E., Acebo, C., Seifer, R., & Carskadon, M. A. (2005, June 18-23). *Activity-based sleep estimation in teens diagnosed with and treated for ADHD: Comparisons with polysomnography and healthy controls* [Conference abstract]. 19th Annual Meeting of the Associated Professional Sleep Societies, LLC (APSS), Denver, CO, United States. https://sleepmeeting.org/wp-content/uploads/2018/10/abstractbook2005.pdf
59. Faraone, S. V., DeSousa, N. J., Komolova, M., Sallee, F. R., Incledon, B., & Wilens, T. E. (2019). Functional impairment in youth with ADHD: Normative data and norm-referenced cutoff points for the before school functioning questionnaire and the parent rating of evening and morning behavior scale, revised. *The Journal of Clinical Psychiatry, 81*(1), 1-13. https://doi.org/10.4088/JCP.19m12956
60. Felt, B., Dore-Stites, D., Well, A., Hassan, F., Chervin, R., & Hoban, T. F. (2010, June 5-9). *Associations between parent report of ADHD symptoms, sleepiness and physical disruptors of sleep* [Conference abstract]. 24th Annual Meeting of the Associated Professional Sleep Societies, LLC (APSS), San Antonia, TX, United States. https://sleepmeeting.org/wp-content/uploads/2018/10/abstractbook2010.pdf
61. Ferri, R., Bruni, O., Novelli, L., Picchietti, M. A., & Picchietti, D. L. (2013). Time structure of leg movement activity during sleep in attention-deficit/hyperactivity disorder and effects of levodopa. *Sleep Medicine, 14*(4), 359-366. https://doi.org/10.1016/j.sleep.2012.12.012
62. Fisher, B. C., Garges, D. M., Yoon, S. Y. R., Maguire, K., Zipay, D., & Gambino, M. (2014). Sex differences and the interaction of age and sleep issues in neuropsychological testing performance across the lifespan in an ADD/ADHD sample from the years 1989 to 2009. *Psychological Reports, 114*(2), 404-438. https://doi.org/10.2466/15.10.PR0.114k23w0
63. Frye, S., Fernandez-Mendoza, J., Calhoun, S. L., Vgontzas, A. N., Liao, D., & Bixler, E. O. (2017, June 3-7). *Role of periodic limb movements during sleep in adolescents with attention deficit hyperactivity disorder: Differential association with internalizing vs. externalizing behaviors* [Conference abstract]. 31st Annual Meeting of the Associated Professional Sleep Societies, LLC (APSS), Boston, MA, United States. https://sleepmeeting.org/wp-content/uploads/2018/10/abstractbook2017.pdf
64. Frye, S. S., Fernandez-Mendoza, J., Calhoun, S. L., Vgontzas, A. N., Liao, D., & Bixler, E. O. (2018). Neurocognitive and behavioral significance of periodic limb movements during sleep in adolescents with attention-deficit/hyperactivity disorder. *Sleep, 41*(10), 1-8. https://doi.org/10.1093/sleep/zsy129
65. Fuller‐Thomson, E., Lewis, D. A, & Agbeyaka, S. K. (2016). Attention‐deficit/hyperactivity disorder casts a long shadow: Findings from a population‐based study of adult women with self‐reported ADHD. *Child: Care, Health and Development, 42*(6), 918-927. https://doi.org/10.1111/cch.12380
66. Furrer, M., Jaramillo, V., Volk, C., Ringli, M., Aellen, R., Wehrle, F. M., Pugin, F., Kurth, S., Brandeis, D., Schmid, M., Jenni, O. G., & Huber, R. (2019). Sleep EEG slow-wave activity in medicated and unmedicated children and adolescents with attention-deficit/hyperactivity disorder. *Translational Psychiatry, 9*(1), 1-8. https://doi.org/10.1038/s41398-019-0659-3
67. Gamble, K. L., May, R. S., Besing, R. C., Tankersly, A. P., & Fargason, R. E. (2013). Delayed sleep timing and symptoms in adults with attention-deficit hyperactivity disorder: A controlled actigraphy study. *Chronobiology International, 30*(4), 598-606. https://doi.org/10.3109/07420528.2012.754454
68. Garbazza, C., Sauter, C., Paul, J., Kollek, J., Dujardin, C., Hackethal, S., Dorn, H., Peter, A., Hansen, M.-L., Manconi, M., Ferri, R., & Danker-Hopfe, H. (2018). Leg movement activity during sleep in adults with attention-deficit/hyperactivity disorder*. Frontiers in Psychiatry, 9*(179), 1-11. https://doi.org/10.3389/fpsyt.2018.00179
69. Gau, S. S.-F. (2006). Prevalence of sleep problems and their association with inattention/hyperactivity among children aged 6–15 in Taiwan*. Journal of Sleep Research, 15*(4), 403-414. https://doi.org/10.1111/j.1365-2869.2006.00552.x
70. Gau, S. S.-F., & Chiang, H.-L. (2009). Sleep problems and disorders among adolescents with persistent and subthreshold attention-deficit/hyperactivity disorders. *Sleep, 32*(5), 671-679. https://doi.org/10.1093/sleep/32.5.671
71. Gau, S. S.-F., Kessler, R. C., Tseng, W.-L., Wu, Y.-Y., Chiu, Y.-N., Yeh, C.-B., & Hwu, H.-G. (2007). Association between sleep problems and symptoms of attention-deficit/hyperactivity disorder in young adults. *Sleep, 30*(2), 195-201. https://doi.org/10.1093/sleep/30.2.195
72. Gau, S. S.-F., Ni, H.-C., Shang, C.-Y., Soong, W.-T., Wu, Y.-Y., Lin, L.-Y., & Chiu, Y.-N. (2010). Psychiatric comorbidity among children and adolescents with and without persistent attention-deficit hyperactivity disorder. *Australian and New Zealand Journal of Psychiatry, 44*(2), 135-143. https://doi.org/10.3109/00048670903282733
73. Gaultney, J. F. (2014). College students with ADHD at greater risk for sleep disorders. *Journal of Postsecondary Education and Disability, 27*(1), 5-18.
74. Gaultney, J. F., Peach, H. D., & Banerjee, M. (2019). Sleep factors may contribute indirectly to association between symptoms of attention-deficit/hyperactivity disorder (ADHD) and impulsivity and future orientation among college students. *Learning Disabilities: A Multidisciplinary Journal, 24*(1), 43-54. https://doi.org/10.18666/LDMJ-2019-V24-I1-9146
75. Gaultney, J. F., Terrell, D. F., & Gingras, J. L. (2005). Parent-reported periodic limb movement, sleep disordered breathing, bedtime resistance behaviors, and ADHD. *Behavioral Sleep Medicine, 3*(1), 32-43. https://doi.org/10.1207/s15402010bsm0301_5
76. Greenhill, L., Puig-Antich, J., Goetz, R., Hanlon, C., & Davies, M. (1983). Sleep architecture and REM sleep measures in prepubertal children with attention deficit disorder with hyperactivity. *Sleep, 6*(2), 91-101. https://doi.org/10.1093/sleep/6.2.91
77. Gruber, R., Sadeh, A. V. I., & Raviv, A. (2000). Instability of sleep patterns in children with attention-deficit/hyperactivity disorder*. Journal of the American Academy of Child and Adolescent Psychiatry, 39*(4), 495-501. https://doi.org/10.1097/00004583-200004000-00019
78. Hansen, B. H., Skirbekk, B., Oerbeck, B., Wentzel-Larsen, T., & Kristensen, H. (2013). Persistence of sleep problems in children with anxiety and attention deficit hyperactivity disorders. *Child Psychiatry and Human Development, 44*(2), 290-304. https://doi.org/10.1007/s10578-012-0325-y
79. Harsh, J. R., Mixon, M. M., Avis, K. T., & LeBourgeois, M. K. (2001, June 5-10). *Sleep, daytime sleepiness, and clinical subtypes of ADHD* [Conference abstract]. 15th Annual Meeting of the Associated Professional Sleep Societies, LLC (APSS) Chicago, IL, United States. https://sleepmeeting.org/wp-content/uploads/2018/10/abstractbook2001.pdf
80. Hazari, N., Joseph, A., Mehta, M., & Sagar, R. (2015). Assessment of sleep disturbances in children with attention-deficit hyperactivity disorder. *Journal of Indian Association for Child and Adolescent Mental Health, 11*, 56-79.
81. Helfer, B. & Asherson, P. (2019, April, 25-28*). Sleepiness and cognitive performance in adults with ADHD: EEG slowing and observer-rated sleepiness* [Conference abstract]. 7th World Congress on ADHD: From Child to Adult Disorder, Lisbon, Portugal. https://link.springer.com/article/10.1007/s12402-019-00295-7
82. Helfer, B., Bozhilova, N., Cooper, R. E., Douzenis, J. I., Maltezos, S., & Asherson, P. (2020). The key role of daytime sleepiness in cognitive functioning of adults with attention deficit hyperactivity disorder. *European Psychiatry, 63*(1), 1-7. https://doi.org/10.1192/j.eurpsy.2020.28.
83. Helwig, J. R. (2011). *Sleep disturbance in children and adolescents with ADHD: Unique effects of medication, ADHD subtype, and comorbid status* [Doctoral Dissertation]. Lehigh University.
84. Hong, G. C. C., Conduit, R., Wong, J., Di Benedetto, M., & Lee, E. (2020). Diet, physical activity, and screen time to sleep better: Multiple mediation analysis of lifestyle factors in school-aged children with and without attention deficit hyperactivity disorder. *Journal of Attention Disorders,* *x*, 1-12. http://doi.org/ 0.1177/108705472094041.
85. Huang, Y.-S., Guilleminault, C., Li, H.-Y., Yang, C.-M., Wu, Y.-Y., & Chen, N.-H. (2006). Attention-deficit/hyperactivity disorder with obstructive sleep apnea: A treatment outcome study. *Sleep Medicine, 8*(1), 18-30. https://doi.org/10.1016/j.sleep.2006.05.016
86. Hysing, M., Heradstveit, O., Harvey, A. G., Nilsen, S. A., Bøe, T., & Sivertsen, B. (2020). Sleep problems among adolescents within child and adolescent mental health services. An epidemiological study with registry linkage. *European Child & Adolescent Psychiatry*, 1-11. https://doi.org/10.1007/s00787-020-01676-4
87. Ibilola, O., & Silva, D. (2017, May 8-10). *The medical and mental health comorbidities of children with ADHD in a sibling control design study* [Conference Abstract]. RACP Congress, Melbourne, Australia. https://onlinelibrary.wiley.com/toc/14401754/2017/53/S3
88. Ito, W., Honda, M., Ueno, T., & Kato, N. (2017, June 3-7). *Subgroup of narcolepsy type 2: Characteristics of sleep variables in hypersomnia patients with attention-deficit hyperactivity disorder (ADHD)* [Conference Abstract]. 31st Anniversary Meeting of the Associated Professional Sleep Societies, Boston, MA, United States. https://sleepmeeting.org/wp-content/uploads/2018/10/abstractbook2017.pdf
89. Ito, W., Komada, Y., Okajima, I., & Inoue, Y. (2017, June 3-7). *Excessive daytime sleepiness in adults with possible attention deficit/hyperactivity disorder (ADHD): A web-based cross-sectional study* [Conference abstract]. RACP Congress, Melbourne, Australia. https://onlinelibrary.wiley.com/toc/14401754/2017/53/S3
90. Ito, W., & Narisawa, H. (2019, April 25-28). *Quantitative electroencephalography in hypersomnia patients with ADHD and narcolepsy patients without ADHD* [Conference abstract]. 7th World Congress on ADHD: From Child to Adult Disorder, Lisbon, Portugal. https://link.springer.com/article/10.1007/s12402-019-00295-7
91. Jackson, D. B., & Vaughn, M. G. (2017). Sleep and preteen delinquency: Is the association robust to ADHD symptomatology and ADHD diagnosis? *Journal of Psychopathology and Behavioral Assessment, 39*(4), 585-595. https://doi.org/10.1007/s10862-017-9610-1
92. Janusis, G. M. (2011). *ADHD symptomology and associated variables in a sample of college students* [Doctoral Dissertation]. University of Rhode Island.
93. Johnson, E. E. H. & Suhr, J. (2021). Self-reported functional impairment in college students: Relationship to noncredible reporting, ADHD, psychological disorders, and other psychological factors. *Journal of Clinical and Experimental Neuropsychology, 43*(4), 399-411. https://doi.org/10.1080/13803395.2021.1935490
94. Kalil Neto, F., & Nunes, M. L. (2016). Evaluation of sleep organization in patients with attention deficit hyperactivity disorder (ADHD) and ADHD as a comorbidity of epilepsy. *Sleep Medicine, 33*, 91-96. https://doi.org/10.1016/j.sleep.2016.08.013
95. Karadag, M., Gokcen, C., Nacarkahya, G., Namiduru, D., Dandil, F., Calisgan, B., & Eroğlu, S. (2019). Chronotypical characteristics and related miR-142-3p levels of children with attention deficit and hyperactivity disorder. *Psychiatry Research, 273*, 235-239. http://doi.org/ 10.1016/j.psychres.2018.12.175
96. Khan, A. U. (1982). Sleep REM latency in hyperkinetic boys. *The American Journal of Psychiatry, 139*(10), 1358-1360.
97. Khurshid, K. A., & Spire, J. (2006, June 17-22). *Attention deficit and hyperactivity disorder (ADHD) in children and sleep disordered breathing* [Conference abstract]. 20th Anniversary Meeting of the Associated Professional Sleep Societies, LLC (APSS), Salt Lake City, UT, United States. https://sleepmeeting.org/wp-content/uploads/2018/10/abstractbook2006.pdf
98. Kidd, A. C. (2009). *Sleep Disorders in Children: A Qualitative Research Study on the Comparison of Behavioral Symptoms Associated with Poor Quality Sleep and ADHD* [Doctoral dissertation]*.* Alliant International University.
99. Kirov, R., Brand, S., Banaschewski, T., & Rothenberger, A. (2017). Opposite impact of REM sleep on neurobehavioral functioning in children with common psychiatric disorders compared to typically developing children. *Frontiers in Psychology, 7*(2059), 1-11. https://doi.org/10.3389/fpsyg.2016.02059
100. Kirov, R., Kinkelbur, J., Banaschewski, T., & Rothenberger, A. (2007). Sleep patterns in children with attention‐deficit/hyperactivity disorder, tic disorder, and comorbidity*. Journal of Child Psychology and Psychiatry, 48*(6), 561-570. https://doi.org/10.1111/j.1469-7610.2007.01729.x
101. Kirov, R., Kinkelbur, J., Heipke, S., Kostanecka‐Endress, T., Westhoff, M., Cohrs, S., Ruther, E., Hajak, G., Banaschewski, T., & Rothenberger, A. (2004). Is there a specific polysomnographic sleep pattern in children with attention deficit/hyperactivity disorder? *Journal of Sleep Research, 13*(1), 87-93. https://doi.org/10.1111/j.1365-2869.2004.00387.x
102. Kirov, R., Uebel, H., Albrecht, B., Banaschewski, T., & Rothenberger, A. (2011, March 11-14). *Two faces of REM sleep in normal and psychopathological development* [Conference abstract]. 19th European Congress of Psychiatry, Vienna, Austria. https://www.cambridge.org/core/journals/european-psychiatry/issue/abstracts-of-the-19th-european-congress-of-psychiatry/A9278FC36749BE8AB9CA21A5E6896FB0
103. Kirov, R., Uebel, H., Albrecht, B., Heckel, L., Banaschewski, T., & Rothenberger, A. (2013, April 6-9). *Increased frequency of SDB and PLMS is associated with lower REM-sleep amount in common child psychopathology and normally developing children* [Conference abstract]. 21st European Congress of Psychiatry, Nice, France. *https://www.cambridge.org/core/journals/european-psychiatry/issue/5289CF2881D6AF40DC55F1080BD99D5F*.
104. Konofal, E., Cortese, S., Marchand, M., Mouren, M.-C., Arnulf, I., & Lecendreux, M. (2007). Impact of restless legs syndrome and iron deficiency on attention-deficit/hyperactivity disorder in children. *Sleep Medicine, 8*(7), 711-715. https://doi.org/10.1016/j.sleep.2007.04.022
105. Kooij, J. J. S., Middelkoop, H. A., van Gils, K., & Buitelaar, J. K. (2001). The effect of stimulants on nocturnal motor activity and sleep quality in adults with ADHD: An open-label case-control study. *The Journal of Clinical Psychiatry, 62*(12), 952-956. https://doi.org/10.4088/JCP.v62n1206
106. Kortesoja, L., Vainikainen, M.-P., Hotulainen, R., Rimpelä, A., Dobewall, H., Lindfors, P., Karvonen, S., & Merikanto, I. (2020). Bidirectional relationship of sleep with emotional and behavioral difficulties: A five-year follow-up of Finnish adolescents. *Journal of Youth and Adolescence, 49*, 1277-1291. https://doi.org/10.1007/s10964-020-01203-3
107. Kwon, S. J., Kim, Y., & Kwak, Y. (2020). Influence of smartphone addiction and poor sleep quality on attention-deficit hyperactivity disorder symptoms in university students: A cross-sectional study. *Journal of American College Health*, 1-7. https://doi.org/10.1080/07448481.2020.1740228
108. Kwon, S. J., Kim, Y., & Kwak, Y. (2020). Relationship of sleep quality and attention deficit hyperactivity disorder symptoms with quality of life in college students. *Journal of American College Health, 68*(5), 536-542. https://doi.org/10.1080/07448481.2019.1583650
109. Lam, L. T., & Yang, L. (2008). Duration of sleep and ADHD tendency among adolescents in China. *Journal of Attention Disorders, 11*(4), 437-444. https://doi.org/10.1177/1087054707299403
110. Langberg, J. M., Breaux, R. P., Cusick, C. N., Green, C. D., Smith, Z. R., Molitor, S. J., & Becker, S. P. (2019). Intraindividual variability of sleep/wake patterns in adolescents with and without attention‐deficit/hyperactivity disorder. *Journal of Child Psychology and Psychiatry,* *60*(11), 1219-1229. https://doi.org/10.1111/jcpp.13082
111. Li, S., Jin, X., Yan, C., Wu, S., Jiang, F., & Shen, X. (2009). Sleep problems in Chinese school-aged children with a parent-reported history of ADHD. *Journal of Attention Disorders, 13*(1), 18-26. https://doi.org/10.1177/1087054708322992
112. Lim, C. G., Ooi, Y. P., Fung, D. S. S., Mahendran, R., & Kaur, A. (2008). Sleep disturbances in Singaporean children with attention deficit hyperactivity disorder. *Annals Academy of Medicine Singapore, 37*(8), 655-61.
113. Lufi, D. & Tzischinsky, O. (2014). The relationships between sensory modulation and sleep among adolescents with ADHD. *Journal of Attention Disorders, 18*(8), 646-653. https://doi.org/10.1177/1087054712457036
114. Lunsford-Avery, J. R., Kollins, S. H., & Krystal, A. D. (2018, June 2-6). *Sleeping at home: Feasibility and tolerability of ambulatory polysomnography for use with adolescents with attention-deficit/hyperactivity disorder* [Conference abstract]. 32nd Annual Meeting of the Associated Sleep Societies, LLC (APSS), Baltimore, MD, United States. https://sleepmeeting.org/wp-content/uploads/2018/10/abstractbook2018.pdf
115. Lunsford-Avery, J., Krystal, A., Carskadon, M. A., & Kollins, S. (2019, December 8-11). *Associations between sleep, neurocognitive performance, and ADHD symptom severity among adolescents* [Conference abstract]. 58th ACNP Annual Meeting, Orlando, FL, United States. https://www.nature.com/collections/dcgjichbhj
116. Lundervold, A. J., Halmøy, A., Jensen, D. A., & Haavik, J. (2019, April 25-28). *The association between insomnia and alcohol consumption in adults with ADHD* [Conference abstract]. 7th World Congress on ADHD: From Child to Adult Disorder, Lisbon, Portugal. https://link.springer.com/article/10.1007/s12402-019-00295-7
117. Lundervold, A. J., Jensen, D. A., & Haavik, J. (2020). Insomnia, Alcohol Consumption and ADHD Symptoms in Adults. *Frontiers in Psychology, 11*(1150), 1-9. https://doi.org/10.3389/fpsyg.2020.01150
118. Madiouni, C., Lopez, R., Gély-Nargeot, M.-C., Lebrun, C., & Bayard, S. (2020). Mind-wandering and sleepiness in adults with attention-deficit/hyperactivity disorder. *Psychiatry Research, 287*(112901), 1-9. https://doi.org/10.1016/j.psychres.2020.112901
119. Marta, R., Guerra, P., Rocha, L., Duarte, A., Lourenco, L., Ventosa, L., Monteiro, J. P., & Fonseca, M. (2010, December 2-4). *Sleep disorders in children with ADHD: A case-control study* [Conference abstract]. 2nd Excellence in Paediatrics Conference, London, United Kingdom. https://onlinelibrary.wiley.com/doi/epdf/10.1111/j.1651-2227.2010.02035.x
120. Mayes, S. D., Calhoun, S., Bixler, E. O., & Vgontzas, A. N. (2009). Sleep problems in children with autism, ADHD, anxiety, depression, acquired brain injury, and typical development. *Sleep Medicine Clinics, 4*(1), 19-25. https://doi.org/10.1016/j.jsmc.2008.12.004
121. Mayes, S., Calhoun, S., Bixler, E., Vgontzas, A., Mahr, F., Hillwig-Garcia, J., Elamir, B., Edhere-Ekezie, L., & Parvin, M. (2009). ADHD subtypes and comorbid anxiety, depression, and oppositional-defiant disorder: Differences in sleep problems. *Journal of Pediatric Psychology, 34*(3), 328-337. https://doi.org/10.1093/jpepsy/jsn083
122. Mayes, S. D., Puzino, K., DiGiovanni, C., & Calhoun, S. L. (2021). Cross-sectional age análisis of sleep problems in 2 to 17 year olds with ADHD combined, ADHD inattentive, or autism. *Journal of Clinical Psychology in Medical Settings*, 1-10. https://doi.org/10.1007/s10880-021-09799-9
123. McGowan, N. M., & Coogan, A. N. (2017, June 23-25). *Social Jetlag and Dysfunctional Circadian Rhythm Entrainment Associate with ADHD Symptoms in Adults* [Conference abstract]. 29th Annual Meeting of the Society for Light Treatment and Biological Rhythms, Berlin, Germany. <https://www-karger-com.kuleuven.ezproxy.kuleuven.be/Article/Pdf/477426>
124. McGowan, N. M., Voinescu, B. I., & Coogan, A. N. (2016). Sleep quality, chronotype and social jetlag differentially associate with symptoms of attention deficit hyperactivity disorder in adults. *Chronobiology international, 33*(10), 1433-1443. https://doi.org/10.1080/07420528.2016.1208214
125. Merikanto, I., Kuula, L., Makkonen, T., Halonen, R., Lahti, J., Heinonen, K., Räikkönen, K., & Pesonen, A. (2019). ADHD symptoms are associated with decreased activity of fast sleep spindles and poorer procedural overnight learning during adolescence. *Neurobiology of Learning and Memory, 157*, 106-113. https://doi.org/10.1016/j.nlm.2018.12.004
126. Merino-Andreu, M., Martinez-Bermejo, A., Casas-Rivero, J., Velazquez-Fraguas, R., & Arcas-Martinez, J. (2008, September 9-13). *Restless legs syndrome is a common finding in children with attention-deficit/hyperactivity disorder* [Conference abstract]. 19th Congress of the European Sleep Research Society, Glasgow, United Kingdom. https://onlinelibrary.wiley.com/doi/epdf/10.1111/j.1365-2869.2008.00690.x
127. Mick, E., Biederman, J., Jetton, J., & Faraone, S. V. (2000). Sleep disturbances associated with attention deficit hyperactivity disorder: The impact of psychiatric comorbidity and pharmacotherapy. *Journal of Child and Adolescent Psychopharmacology, 10*(3), 223-231. https://doi.org/10.1089/10445460050167331
128. Migliarese, G., Torriero, S., Gesi, C., Venturi, V., Reibman, Y., Cerveri, G., Viganó, V., Decaroli, G., Ricciardelli, P., & Mencacci, C. (2020). Sleep quality among adults with attention deficit hyperactivity disorder or autism spectrum disorder: which is the role of gender and chronotype?. *Sleep Medicine, 76*, 128-133. https://doi.org/10.1016/j.sleep.2020.10.015
129. Miniksar, D. Y. & Özdemir, M. (2021). Sleep quality in children and adolescents with attention-deficit and hyperactivity disorder. *Archives de Pédiatrie, 28*(8), 668-676. https://doi.org/10.1016/j.arcped.2021.09.017
130. Montagni, I., Qchiqach, S., Pereira, E., Tully, P. J., & Tzourio, C. (2020). Sex-specific associations between sleep and mental health in university students: A large cross-sectional study. *Journal of American College Health, 68*(3), 278-285. https://doi.org/10.1080/07448481.2018.1546183
131. Moreau, V., Rouleau, N., & Morin, C. M. (2014). Sleep of children with attention deficit hyperactivity disorder: Actigraphic and parental reports. *Behavioral Sleep Medicine, 12*(1), 69-83. https://doi.org/10.1080/15402002.2013.764526
132. Nakatani, M., Okada, S., Shimizu, S., Mohri, I., Ohno, Y., Taniike, M., & Makikawa, M. (2013, July 3-7). *Body movement analysis during sleep for children with ADHD using video image processing* [Conference abstract]. 40th Annual International Conference of the IEEE Engineering in Medicine and Biology Society (EMBC), Osaka, Japan. https://ieeexplore.ieee.org/xpl/conhome/6596169/proceeding
133. Navarro-Soria, I., Real-Fernández, M., Juárez-Ruiz de Mier, R., Costa-López, B., Sánchez, M., & Lavigne, R. (2021). Consequences of confinement due to COVID-19 in Spain on anxiety, sleep and executive functioning of children and adolescents with ADHD. *Sustainability, 13*(5), 1-17. https://doi.org/10.3390/su13052487
134. Niers, T., Tulen, J. H., Vegt, R., & Hengeveld, M. W. (2006, October 25-29). *Motor activity during sleep, sleep quality and symptom severity in adults with ADHD or Tourette’s disorder* [Conference abstract]. 46th Annual Meeting of the Society for Psychophysiological Research, Vancouver, Canada. https://cdn.ymaws.com/sprweb.org/resource/resmgr/pastmeetingdocs/2007program.pdf
135. O'Brien, L. M., Ivanenko, A., Crabtree, V. M., Holbrook, C. R., Bruner, J. L., Klaus, C. J., & Gozal, D. (2003). Sleep disturbances in children with attention deficit hyperactivity disorder. *Pediatric Research, 54*(2), 237-243. https://doi.org/10.1203/01.PDR.0000072333.11711.9A
136. O'Brien, L. M., Ivanenko, A., Crabtree, V. M., Holbrook, C. R., Bruner, J. L., Klaus, C. J., & Gozal, D. (2003). The effect of stimulants on sleep characteristics in children with attention deficit/hyperactivity disorder. *Sleep Medicine, 4*(4), 309-316. https://doi.org/10.1016/S1389-9457(03)00071-6
137. O'Brien, E., & Mindell, J. A. (2006, June 17-22). *Sleep and daytime functioning in adolescents with ADHD or a learning disability* [Conference abstract]. 20th Anniversary Meeting of the Associated Professional Sleep Societies, LLC (APSS), Salt Lake City, UT, United States. https://sleepmeeting.org/wp-content/uploads/2018/10/abstractbook2006.pdf
138. Ogundele, M. O. (2018, March 13-15). *Management of sleep difficulties among a cohort of children with ADHD in a Scottish local authority* [Conference abstract]. Annual Conference of the Royal College of Paediatrics and Child Health, Glasgow, Scotland. https://adc.bmj.com/content/103/Suppl_1
139. Owens, J., Sangal, R. B., Sutton, V. K., Bakken, R., Allen, A. J., & Kelsey, D. (2009). Subjective and objective measures of sleep in children with attention-deficit/hyperactivity disorder*. Sleep Medicine, 10*(4), 446-456. https://doi.org/10.1016/j.sleep.2008.03.013
140. Park, S., Cho, M. J., Chang, S. M., Jeon, H. J., Cho, S.-J., Kim, B.-S., Bae, J. N., Wang, H.-R., Ahn, J. H., & Hong, J. P. (2011). Prevalence, correlates, and comorbidities of adult ADHD symptoms in Korea: Results of the Korean epidemiologic catchment area study. *Psychiatry Research, 186*(2-3), 378-383. https://doi.org/10.1016/j.psychres.2010.07.047
141. Philip, P., Canel, A., Micoulaud-Franchi, J.-A., Sagaspe, P., & Bioulac, S. (2015, June 6-10*). Sleepiness, distraction, attention deficit hyperactivity symptoms and accidental risk in a large group of regular registered highway drivers* [Conference abstract]. 29th Annual Meeting of the Associated Professional Sleep Societies, LLC (APSS), Seattle, WA, United States. https://sleepmeeting.org/wp-content/uploads/2018/10/abstractbook2015.pdf
142. Philip, P., Micoulaud-Franchi, J.-A., Lagarde, E., Taillard, J., Canel, A., Sagaspe, P., & Bioulac, S. (2015). Attention deficit hyperactivity disorder symptoms, sleepiness and accidental risk in 36140 regularly registered highway drivers. *PloS One, 10*(9), 1-14. https://doi.org/10.1371/journal.pone.0138004
143. Philipsen, A., Feige, B., Hesslinger, B., Ebert, D., Carl, C., Hornyak, M., Lieb, K., Voderholzer, U., & Riemann, D. (2005). Sleep in adults with attention-deficit/hyperactivity disorder: A controlled polysomnographic study including spectral analysis of the sleep EEG. *Sleep, 28*(7), 877-884. https://doi.org/10.1093/sleep/28.7.877
144. Picchietti, D. L., Underwood, D. J., Farris, W. A., Walters, A. S., Shah, M. M., Dahl, R. E., Trubnick, L. J., Bertocci, M. A., Wagner, M., & Hening, W. A. (1999). Further studies on periodic limb movement disorder and restless legs syndrome in children with attention‐deficit hyperactivity disorder. *Movement Disorders, 14*(6), 1000-1007. https://doi.org/10.1002/1531-8257(199911)14:6<1000::AID-MDS1014>3.0.CO2-P
145. Poirier, A., & Corkum, P. (2018). Night-to-night variability of sleep in children with ADHD and typically developing controls. *Journal of Attention Disorders, 22*(10), 942-946. https://doi.org/10.1177/1087054715575065
146. Prehn-Kristensen, A., Göder, R., Fischer, J., Wilhelm, I., Seeck-Hirschner, M., Aldenhoff, J., & Baving, L. (2011). Reduced sleep-associated consolidation of declarative memory in attention-deficit/hyperactivity disorder. *Sleep Medicine, 12*(7), 672-679. https://doi.org/10.1016/j.sleep.2010.10.010
147. Reimer, B., D'Ambrosio, L. A., Coughlin, J. F., Fried, R., & Biederman, J. (2007). Task-induced fatigue and collisions in adult drivers with attention deficit hyperactivity disorder. *Traffic Injury Prevention, 8*(3), 290-299. https://doi.org/10.1080/15389580701257842
148. Ricci, A., He, F., Calhoun, S. L., Fang, J., Vgontzas, A. N., Liao, D., Bixler, E. O., & Fernandez-Mendoza, J. (2022). Evidence of a maturational disruption in non-rapid eye movement sleep slow wave activity in youth with attention-deficit/hyperactivity, learning and internalizing disorders. *Sleep Medicine, 90*, 230-237. https://doi.org/10.1016/j.sleep.2022.01.026
149. Ringli, M., Souissi, S., Kurth, S., Brandeis, D., Jenni, O. G., & Huber, R. (2013). Topography of sleep slow wave activity in children with attention-deficit/hyperactivity disorder. *Cortex, 49*(1), 340-347. https://doi.org/10.1016/j.cortex.2012.07.007
150. Rogers, D. C., Dittner, A. J., Rimes, K. A., & Chalder, T. (2017). Fatigue in an adult attention deficit hyperactivity disorder population: A trans‐diagnostic approach*. British Journal of Clinical Psychology, 56*(1), 33-52. https://doi.org/10.1111/bjc.12119
151. Roy, M., De Zwaan, M., Tuin, I., Philipsen, A., Brähler, E., & Müller, A. (2018). Association between restless legs syndrome and adult ADHD in a German community-based sample. *Journal of Attention Disorders, 22*(3), 300-308. https://doi.org/10.1177/1087054714561291
152. Saito, Y., Kaga, Y., Nakagawa, E., Okubo, M., Kohashi, K., Omori, M., Fukuda, A., & Inagaki, M. (2019). Association of inattention with slow-spindle density in sleep EEG of children with attention deficit-hyperactivity disorder. *Brain and Development, 41*(9), 751-759. https://doi.org/10.1016/j.braindev.2019.05.004
153. Saletin, J. M., Coon, W. G., & Carskadon, M. A. (2017). Stage 2 sleep EEG sigma activity and motor learning in childhood ADHD: A pilot study. *Journal of Clinical Child and Adolescent Psychology, 46*(2), 188-197. https://doi.org/10.1080/15374416.2016.1157756
154. San Mauro Martín, I., Blumenfeld Olivares, J. A., Garicano Vilar, E., Echeverry López, M., García Bernat, M., Quevedo Santos, Y., Blanco López, M., Elortegui Pascual, P., Borregon Rivilla, E., & Rincón Barrado, M. (2018). Nutritional and environmental factors in attention-deficit hyperactivity disorder (ADHD): A cross-sectional study. *Nutritional Neuroscience, 21*(9), 641-647. https://doi.org/10.1080/1028415X.2017.1331952
155. Sawyer, A. C. P., Clark, C. R., Keage, H. A. D., Moores, K. A., Clarke, S., Kohn, M. R., & Gordon, E. (2008). Cognitive and electroencephalographic disturbances in children with attention-deficit/hyperactivity disorder and sleep problems: New insights. *Psychiatry Research, 170*(2), 183-191. https://doi.org/10.1016/j.psychres.2008.10.026
156. Schlarb, A., & Gruenwald, J. (2018, September 25-28*). Insomnia, nightmares and daytime sleepiness in university students with ADHD* [Conference abstract]. 24th Congress of the European Sleep Research Society, Basel, Switzerland. https://onlinelibrary.wiley.com/doi/epdf/10.1111/jsr.12751
157. Schredl, M., Alm, B., & Sobanski, E. (2007). Sleep quality in adult patients with attention deficit hyperactivity disorder (ADHD). *European Archives of Psychiatry and Clinical Neuroscience, 257*(3), 164-168. https://doi.org/10.1007/s00406-006-0703-1
158. Schredl, M., Bumb, J. M., Alm, B., & Sobanski, E. (2017). Nightmare frequency in adults with attention-deficit hyperactivity disorder. *European Archives of Psychiatry and Clinical Neuroscience, 267*(1), 89-92. https://doi.org/10.1007/s00406-016-0686-5
159. Shen, C., Luo, Q., Chamberlain, S. R., Morgan, S., Romero-Garcia, R., Du, J., Zhao, X., Touchette, E., Montplaisir, J., Vitaro, F., Boivin, M., Tremblay, R. E., Zhao, X.-M., Robaey, P., Feng, J. & Sahakian, B. J. (2020). What is the link between attention-deficit/hyperactivity disorder and sleep disturbance? A multimodal examination of longitudinal relationships and brain structure using large-scale population-based cohorts. *Biological Psychiatry,* 1-11. https://doi.org/10.1016/j.biopsych.2020.03.010
160. Sivertsen, B., Harvey, A. G., Pallesen, S., & Hysing, M. (2015). Mental health problems in adolescents with delayed sleep phase: Results from a large population‐based study in Norway. *Journal of Sleep Research, 24*(1), 11-18. https://doi.org/10.1111/jsr.12254
161. Sobanski, E., Alm, B., Hennig, O., Riemann, D., Feige, B., & Schredl, M. (2016). Daytime sleepiness in adults with ADHD. *Journal of Attention Disorders, 20*(12), 1023-1029. https://doi.org/10.1177/1087054714529456
162. Sobanski, E., Schredl, M., Kettler, N., & Alm, B. (2008). Sleep in adults with attention deficit hyperactivity disorder (ADHD) before and during treatment with methylphenidate: A controlled polysomnographic study*. Sleep, 31*(3), 375-381. https://doi.org/10.1093/sleep/31.3.375
163. Spera, V., Maiello, M., Pallucchini, A., Novi, M., Elefante, C., De Dominicis, F., Palagini, L., Biederman, J., & Perugi, G. (2020). Adult attention-deficit hyperactivity disorder and clinical correlates of delayed sleep phase disorder. *Psychiatry Research, 291*, 1-6. https://doi.org/10.1016/j.psychres.2020.113162
164. Stein, M. A. (1999). Unravelling sleep problems in treated and untreated children with ADHD. *Journal of Child and Adolescent Psychopharmacology, 9*(3), 157-168. https://doi.org/10.1089/cap.1999.9.157
165. Stein, D., Pat-Horenczyk, R., Blank, S., Dagan, Y., Barak, Y., & Gumpel, T. P. (2002). Sleep disturbances in adolescents with symptoms of attention-deficit/hyperactivity disorder. *Journal of Learning Disabilities, 35*(3), 268-275. https://doi.org/10.1177/002221940203500308
166. Stephens, R. J. (2001). *REM sleep and aggressive behaviour in children with Tourette's syndrome (TS), attention deficit hyperactivity disorder (ADHD), and comorbid TS and ADHD* [Doctoral dissertation], University of Toronto.
167. Stephens, R. J., Chung, S. A., Jovanovic, D., Guerra, R., Stephens, B., Sandor, P., & Shapiro, C. M. (2013). Relationship between polysomnographic sleep architecture and behavior in medication-free children with TS, ADHD, TS and ADHD, and controls*. Journal of Developmental and Behavioral Pediatrics, 34*(9), 688-696. https://doi.org/10.1097/DBP.0000000000000012
168. Surman, C. B. H., Adamson, J. J., Petty, C., Biederman, J., Kenealy, D. C., Levine, M., Mick, E., & Faraone, S. V. (2009). Association between attention-deficit/hyperactivity disorder and sleep impairment in adulthood: Evidence from a large controlled study*. The Journal of Clinical Psychiatry, 70*(11), 1523-1529. https://doi.org/10.4088/JCP.08m04514
169. Taillard, J., Sagaspe, P., Bioulac, S., Micoulaud-Franchi, J.-A., & Philip, P. (2017, December). *Chronotype, chronic sleep restriction, social jet lag and ADHD symptoms in 18436 active adults* [Conference abstract]. World Association of Sleep Medicine, Prague, Czech Republic. https://www.sciencedirect.com/journal/sleep-medicine/vol/40/suppl/S1
170. Thoma, V. K., Schulz-Zhecheva, Y., Oser, C., Fleischhaker, C., Biscaldi, M., & Klein, C. (2020). Media use, sleep quality, and ADHD symptoms in a community sample and a sample of ADHD patients aged 8 to 18 years. *Journal of Attention Disorders, 24*(4), 576-589. https://doi.org/10.1177/1087054718802014
171. Tidwell, T. J., Hovinga, C. A., Castillo, S. M., & Kang, J. B. (2016, June 11-15). *Attention deficit/hyperactivity disorder treatment and periodic limb movements in sleep* [Conference abstract]. 30th Anniversary Meeting of the Associated Professional Sleep Societies, Denver, CO, United States. https://sleepmeeting.org/wp-content/uploads/2018/10/abstractbook2016.pdf
172. Tomás-Vila, M., Miralles Torres, A., Beseler Soto, B., Revert Gomar, M., Sala Langa M., & Uribelarrea Sierra, A. (2008). Attention-deficit/hyperactivity disorder and sleep disturbances. Results of an epidemiological study in schoolchildren in Gandia, Spain. *Anales de pediatria*, *69*(3), 251-257. https://doi.org 10.1157/13125820
173. Tonetti, L., Conca, A., Giupponi, G., & Natale, V. (2017). Circadian pattern of motor activity in adults with attention-deficit/hyperactivity disorder. *Chronobiology International, 34*(6), 802-807. https://doi.org/10.1080/07420528.2017.1309660
174. Tonetti, L., Conca, A., Giupponi, G., Filardi, M., & Natale, V. (2018). Circadian activity rhythm in adult attention-deficit hyperactivity disorder. *Journal of Psychiatric Research, 103*, 1-4. https://doi.org/10.1016/j.jpsychires.2018.05.002
175. Tsai, F.-J., Chiang, H.-L., Lee, C.-M., Gau, S. S.-F., Lee, W.-T., Fan, P.-C., Wu, Y.-Y., & Chiu, Y.-N. (2012). Sleep problems in children with autism, attention-deficit hyperactivity disorder, and epilepsy. *Research in Autism Spectrum Disorders, 6*(1), 413-421. https://doi.org/10.1016/j.rasd.2011.07.002
176. Tsai, J.-D., Wang, I.-C., Chen, H.-J., Sheu, J.-N., Li, T.-C., Tsai, H. J., & Wei, C.-C. (2017). Trend of nocturnal enuresis in children with attention deficit/hyperactivity disorder: A nationwide population-based study in Taiwan. *Journal of Investigative Medicine, 65*(2), 370-375. https://doi.org/10.1136/jim-2016-000223
177. Tsuji, Y. & Takeda, T. (2018, July 12-13). *Prediction of sleep disturbances depends on the intensity of ADHD symptoms: Logistic regression analysis* [Conference abstract]. 9th Congress of Asian Sleep Research Society, Sapporo, Japan. https://link.springer.com/content/pdf/10.1007/s41105-018-0176-4.pdf
178. Vaidyanathan, S., Shah, H., & Gayal, T. (2016). Sleep disturbances in children with attention–deficit/hyperactivity disorder (ADHD): Comparative study with healthy siblings. *Journal of the Canadian Academy of Child and Adolescent Psychiatry, 25*(3), 145.
179. Van Andel, E., Ten Have, M., Bijlenga, D., Beekman, A. T. F., De Graaf, R., & Kooij, J. J. S. (2020). Combined impact of ADHD and insomnia symptoms on quality of life, productivity, and health care use in the general population. *Psychological Medicine,* 1-12. <https://doi.org/10.1017/S0033291720001592>
180. Van Veen, M. M., Kooij, J. J. S., Boonstra, A. M., Gordijn, M. C. M., & Van Someren, E. J. W. (2010). Delayed circadian rhythm in adults with attention-deficit/hyperactivity disorder and chronic sleep-onset insomnia. *Biological Psychiatry, 67*(11), 1091-1096. https://doi.org/10.1016/j.biopsych.2009.12.032
181. Vélez-Galarraga, R., Guillen-Grima, F., Crespo-Eguílaz, N., & Sánchez-Carpintero, R. (2016). Prevalence of sleep disorders and their relationship with core symptoms of inattention and hyperactivity in children with attention-deficit/hyperactivity disorder. *European Journal of Paediatric Neurology, 20*(6), 925-937. https://doi.org/10.1016/j.ejpn.2016.07.004
182. Virring, A., Lambek, R., Jennum, P. J., Møller, L. R., & Thomsen, P. H. (2017). Sleep problems and daily functioning in children with ADHD: An investigation of the role of impairment, ADHD presentations, and psychiatric comorbidity. *Journal of Attention Disorders, 21*(9), 731-740. https://doi.org/10.1177/1087054714542001
183. Vogel, S. W. N., Bijlenga, D., Benjamins, J., Beekman, A. T. F., Kooij, J. J. S., & Van Someren, E. J. W. (2016, September 13-16). *Sleep disturbances and attention-deficit/hyperactivity disorder symptoms in adult participants of the Netherlands Sleep Registry* [Conference abstract]. 23rd Congress of the European Sleep Research Society, Bologna, Italy. https://onlinelibrary.wiley.com/doi/epdf/10.1111/jsr.12446
184. Vogel, S. W. N., Bijlenga, D., Tanke, M., Bron, T. I., Van der Heijden, K. B., Swaab, H., Beekman, A. T. F., & Sandra Kooij, J. J. S. (2015). Circadian rhythm disruption as a link between attention-deficit/hyperactivity disorder and obesity? *Journal of Psychosomatic Research, 79*(5), 443-450. https://doi.org/10.1016/j.jpsychores.2015.10.002
185. Voinescu, B., Szentagotai, A., & Thome, J. (2011, October). *Sleep disturbance and attention deficit/hyperactivity symptoms in young adults* [Conference abstract]. World Sleep Conference, Kyoto, Japan. https://link.springer.com/content/pdf/10.1111/j.1479-8425.2011.00518.x.pdf
186. Walker, B. M. (2017). *An Examination of the Relationship Between Sleep and Parental Distress in Medicated and Partially-Medicated Children With ADHD* [Doctoral dissertation]*.* Howard University.
187. Waxmonsky, J. G., Mayes, S. D., Calhoun, S. L., Fernandez-Mendoza, J., Waschbusch, D. A., Bendixsen, B. H., & Bixler, E. O. (2017). The association between disruptive mood dysregulation disorder symptoms and sleep problems in children with and without ADHD. *Sleep Medicine, 37*, 180-186. https://doi.org/10.1016/j.sleep.2017.02.006
188. Weibel, S., Jermann, F., Weiner, L., Nicastro, R., Ardu, S., Pham, E., Hasler, R., Dayer, A., Prada, P., & Perroud, N. (2017). Insomnia in adult attention-deficit/hyperactivity disorder: A comparison with borderline personality disorder population in a clinical setting and control participants. *Comprehensive Psychiatry, 76*, 119-128. <https://doi.org/10.1016/j.comppsych.2017.04.009>
189. Weiner, L., Ossola, P., Causin, J. B., Kraemer, C., Bertschy, G., & Weibel, S. (2018, March 3-6). *Racing thoughts in adults with ADHD: A neglected symptom associated with mood instability and insomnia* [Conference abstract]. 26th European Congress of Psychiatry, Nice, France. https://www.cambridge.org/core/services/aop-cambridge-core/content/view/B2C237FD3C2963992E5AE741696A336D/S0924933800017946a.pdf/eposter_walk.pdf
190. Wynchank, D. S., Bijlenga, D., Lamers, F., Bron, T. I., Winthorst, W. H., Vogel, S. W. N., Penninx, B. W., Beekman, A. T. F., & Kooij, J. J. S. (2016). ADHD, circadian rhythms and seasonality. *Journal of Psychiatric Research, 81*, 87-94. https://doi.org/10.1016/j.jpsychires.2016.06.018
191. Wynchank, D., Ten Have, M., Bijlenga, D., Penninx, B. W., Beekman, A. T. F., Lamers, F., de Graaf, R., & Kooij, J. J. S. (2018). The association between insomnia and sleep duration in adults with attention-deficit hyperactivity disorder: Results from a general population study. *Journal of Clinical Sleep Medicine, 14*(3), 349-357. https://doi.org/10.5664/jcsm.6976
192. Yang, L.-K., Shang, C.-Y., & Gau, S. S.-F. (2011). Psychiatric comorbidities in adolescents with attention-deficit hyperactivity disorder and their siblings. *The Canadian Journal of Psychiatry, 56*(5), 281-292. https://doi.org/10.1177/070674371105600507
193. Yemula, C., Musgrave, E., Ajmal, S., Khan, A., Banerjee, T., Sesham, R., & Jainer, R. (2019, April 25-28). *A clinic-based survey of teenagers with and without ADHD to understand their sleep habits, impact of poor sleep and whether gender differences exist* [Conference abstract]. 7th World Congress on ADHD: From Child to Adult Disorder, Lisbon, Portugal. https://link.springer.com/content/pdf/10.1007/s12402-019-00295-7.pdf
194. Yeom, C.-W., Oh, G. H., Jung, S., Moon, J. Y., Son, K.-L., Kim, W.-H., Jung, D., Baik, M., Shim, E.-J., Moon, H., & Hahm, B. J. (2020). Prevalence and comorbidities of adult ADHD in male military conscripts in Korea: Results of an epidemiological survey of mental health in Korean military service. *Psychiatry Research, 293*(113401), 1-7. https://doi.org/10.1016/j.psychres.2020.113401
195. Zarei, S., Chung, S. A., Shahid, A., Martin, J., & Shapiro, C. M. (2016, June 11-15). *Is there a connection between ADHD-like symptoms and disturbed sleep in children?* [Conference abstract]. 30th Anniversary Meeting of the Associated Professional Sleep Societies, LLC (APSS), Denver, CO, United States. https://sleepmeeting.org/wp-content/uploads/2018/10/abstractbook2016.pdf
196. Zhao, Y., Jiang, Z., Guo, S., Wu, P., Lu, Q., Xu, Y., Liu, L., Su, S., Shi, L., Que, J., Sun, Y., Sun, Y., Deng, J., Meng, S., Yan, W., Yuan, K., Sun, S., Yang, L., Ran, M. … & Shi, J. (2021). Association of symptoms of attention deficit and hyperactivity with problematic internet use among university students in Wuhan, China during the COVID-19 pandemic. *Journal of Affective Disorders, 286*, 220-227. https://doi.org/10.1016/j.jad.2021.02.078

**Table S3**

*Sensitivity analyses*

|  |  |  |  |  |  |  | Heterogeneity | |
| --- | --- | --- | --- | --- | --- | --- | --- | --- |
|  | ADHD (n) | Control (n) | SMD  (95% CI) | z | p | Effect size | I^2^ | p |
| Subjective sleep parameters | | | | | | | | |
| Age |  |  |  |  |  |  |  |  |
| TST (2) | 84 | 1954 | .79 (.00-1.57) | 1.97 | .05 | Large | 91% | .001 |
| SOL (3) | 136 | 2004 | .51 (.28-.73) | 4.43 | < .001 | Medium | 22% | .28 |
| Medication |  |  |  |  |  |  |  |  |
| TST (2) | 220 | 204 | .17 (-.11-.44) | 1.18 | .24 |  | 44% | .18 |
| TST week (2) | 220 | 204 | .15 (-.22-.53) | .79 | .43 |  | 69% | .07 |
| TST weekend (2) | 220 | 204 | .16 (-.03-.35) | 1.67 | .09 |  | 0% | .59 |
| Bedtime (2) | 220 | 204 | -.15 (-.34-.05) | 1.49 | .14 |  | 0% | 1.00 |
| Bedtime week (2) | 220 | 204 | -.14 (-.33-.05) | 1.41 | .16 |  | 0% | .56 |
| Bedtime weekend (2) | 220 | 204 | .10 (-.32-.52) | .47 | .64 |  | 74% | .05 |
|  | | | | | | | | |
| Sleep problems | | | | | | | | |
| Age |  |  |  |  |  |  |  |  |
| Insomnia (2) | 131 | 541 | .16 (-.03-.36) | 1.62 | .11 |  | 0% | .46 |
| Medication |  |  |  |  |  |  |  |  |
| Daytime sleepiness (2) | 220 | 204 | -.04 (-.86-.78) | .11 | .92 |  | 93% | <.001 |
| Sleep disturbances (2) | 342 | 243 | .69 (.52-.86) | 8.00 | < .001 | Medium | 0% | .44 |
